# Vision toolkit part 3. Scanpaths and derived representations for gaze behavior characterization: a review

**DOI:** 10.3389/fphys.2025.1721768

**Published:** 2026-01-27

**Authors:** Quentin Laborde, Axel Roques, Allan Armougum, Nicolas Vayatis, Ioannis Bargiotas, Laurent Oudre

**Affiliations:** 1 Université Paris Saclay, Université Paris Cité, ENS Paris Saclay, CNRS, SSA, INSERM, Centre Borelli, Gif-sur-Yvette, France; 2 Technologies Department, Innovation and Research, SNCF, Saint Denis, France; 3 Training and Simulation, Thales AVS France, Osny, France; 4 Inria, CIAMS, Université Paris-Saclay, Gif-sur-Yvette, France

**Keywords:** eye-tracking, recurrence quantification analysis, saliency map, scanpath, scanpath comparison

## Abstract

Scanpath analysis provides a powerful window into visual behavior by jointly capturing the spatial organization and temporal dynamics of gaze. By linking perception, cognition, and oculomotor control, scanpaths offer rich insights into how individuals explore visual scenes and accomplish task goals. Despite decades of research, however, the field remains methodologically fragmented, with a wide diversity of representations and comparison metrics that complicate interpretation and methodological choice. This article reviews computational approaches for the characterization and comparison of scanpaths, with an explicit focus on their underlying assumptions, interpretability, and practical implications. We first survey representations and metrics designed to describe individual scanpaths, ranging from geometric descriptors and spatial density representations to more advanced approaches such as attention maps, recurrence quantification analysis, and symbolic string encodings that capture temporal regularities and structural patterns. We then review methods for comparing scanpaths across observers, stimuli, or tasks, including point-mapping metrics, elastic alignment techniques, string-edit distances, saliency-based measures, and hybrid approaches integrating spatial and temporal information. Across these methods, we highlight their respective strengths, limitations, and sensitivities to design choices such as discretization, spatial resolution, and temporal weighting. Rather than promoting a single optimal metric, this review emphasizes scanpath analysis as a family of complementary tools whose relevance depends on the research question and experimental context. Overall, this work aims to provide a unified conceptual framework to guide methodological selection, foster reproducibility, and support the meaningful interpretation of gaze dynamics across disciplines.

## 1 Introduction

Understanding how humans explore their visual environment has been a central topic in *eye-tracking research* for nearly a century. The term *scanpath* was first introduced by Noton and Stark (1971a) and Noton and Stark (1971b), who proposed that an internal cognitive representation guides both visual perception and the associated mechanism of active eye movements in a top-down manner. Their pioneering work suggested that gaze behavior reflects deeper cognitive processes such as expectations, memory, and task goals. This groundbreaking idea is considered one of the most influential theories in the study of vision and eye movements. However, these key concepts were also foreshadowed in earlier classic works on eye movements. In particular, Yarbus (1967b) demonstrated that gaze patterns vary systematically with the observer’s instructions: when viewing the same painting under distinct task sets, participants produced markedly different trajectories. These findings revealed that fixation locations, their temporal ordering, and the overall structure of the scanpath depend jointly on stimulus properties and the observer’s mental state. Subsequent influential contributions to scanpath analysis include the work of Choi et al. (1995), who introduced string-based representations for visual search, as well as studies by Zangemeister et al. (1995a) and Zangemeister et al. (1995b), which demonstrated the existence of global scanpath strategies and high-level oculomotor control in both healthy observers and patients with visual field defects.

**FIGURE 1:**
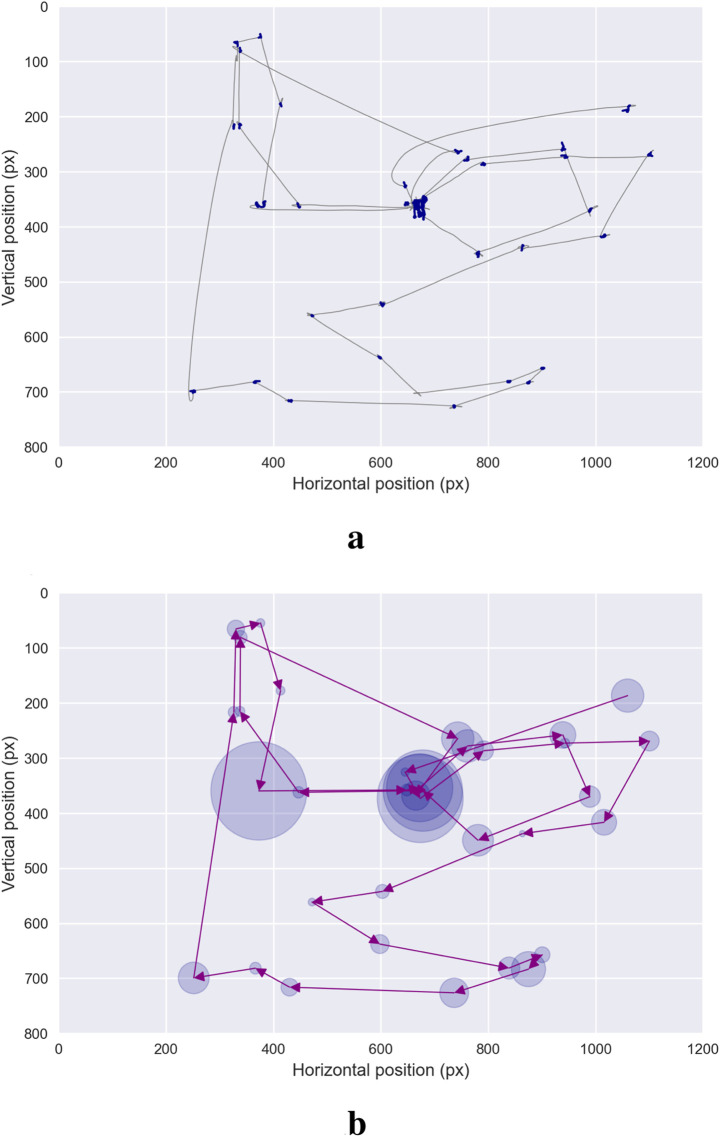
Scanpath. This figure illustrates a commonly used representation of scanpath trajectories. Fixations are first extracted from raw gaze data using binary segmentation algorithms — **(a)** The scanpath is then visualized **(b)** — with fixations represented at the centroid of their spatial coordinates. The temporal aspect of fixations is depicted using blue circles, with the radius proportional to the fixation duration. Purple lines connect successive fixations, representing saccades—the non-linear trajectory of saccades is thus abandoned in favor of a simplified representation.

For the purposes of this review, we define a *scanpath* as a sequence of successive eye fixations, each specified by its spatial location—horizontal and vertical coordinates—and its associated duration. The process for constructing scanpath trajectories generally begins by segmenting raw gaze recordings into slow—fixation—and fast—saccadic—phases. After segmentation, slow phases are grouped into fixation events, while saccades are collapsed into transition events between fixations, thereby producing scanpath time series. It is important to emphasize that this abstraction captures the essential dynamics of visual exploration: fixations represent moments of relative perceptual stability, whereas saccades indicate shifts of attention between loci of interest. Figure 1 provides a schematic representation of this transformation from raw gaze signals to scanpath trajectories.

The classic *scanpath theory* posits that scanpaths are predominantly *top-down* processes, driven by an observer’s mental model. In this view, cognitive goals and intentions dictate fixation locations, adapting to the task at hand. However, alternative perspectives, such as visual saliency models, emphasize the role of *bottom-up* influences, wherein low-level stimulus properties—*e.g.*, contrast, color, and motion—capture attention and guide eye movements. These models argue that salient features in the visual field dictate gaze trajectories, with cognitive influences acting secondarily. One key limitation of scanpath theory in its strongest form is its inability to fully explain variability in eye movements across different observers and tasks. Similarly, a purely *bottom-up* saliency model also struggles to account for the diversity in gaze patterns during repeated exposures to the same visual stimulus.

Over recent decades, considerable debate has revolved around the interplay between *top-down* and *bottom-up* mechanisms in the control of visual attention (Theeuwes, 2010). Whereas early frameworks tended to treat these mechanisms as competing sources of guidance, more recent accounts emphasize a dynamic and interactive process unfolding over multiple timescales. According to this view, initial fixations are predominantly driven by *bottom-up* salience—reflecting local stimulus properties such as contrast, motion, or color—while later stages increasingly reflect *top-down* influences related to task goals, expectations, prior knowledge, and learned attentional sets (Hochstein and Ahissar, 2002; VanRullen and Koch, 2003; Wolfe, 2021). These influences interact through recurrent processing loops linking higher-order cortical areas with early visual regions, enabling cognitive goals to progressively reshape fixation patterns during exploration. Contemporary computational models likewise implement hybrid architectures in which salience, goal-driven priority maps, and learned attentional biases jointly contribute to fixation selection (Mengers et al., 2025). Together, these findings converge toward a multifactorial account in which bottom-up signals dominate initial orienting but are rapidly integrated with feedback mechanisms that incorporate task demands, contextual expectations, and experience-driven biases.

Computational characterization of scanpaths is methodologically challenging because it requires capturing sequential dependencies, spatial distributions, and temporal dynamics. Since the early work of Noton and Stark, the field has grown substantially, producing a diverse array of approaches (Anderson et al., 2013; Brandt and Stark, 1997; Burmester and Mast, 2010; Foulsham et al., 2012; Foulsham and Underwood, 2008; Johansson et al., 2006; Shepherd et al., 2010). This review of scanpath analysis and representations is organized into two main sections. First, we outline the geometric and descriptive characteristics of scanpaths, including representations derived from fixation sequences and quantitative measures that capture the spatial and temporal properties of fixation trajectories. Second, we examine the extensive body of work devoted to comparing scanpath trajectories, a key aspect of gaze dynamics research.

This article is the third contribution in an ongoing series of methodological reviews dedicated to the analysis of oculomotor signals and gaze trajectories. The first article, published in *Frontiers in Physiology* (Laborde et al., 2025b), synthesizes current knowledge on canonical eye movements, with particular emphasis on the differences between controlled laboratory settings and naturalistic viewing conditions. The second article (Laborde et al., 2025a) reviews segmentation algorithms and oculomotor features that enable the reliable identification and characterization of fixations, saccades, and smooth pursuits. The present work focuses on the *representations and metrics* used to characterize scanpaths, as well as on the methods for comparing scanpaths across stimuli, observers, or tasks.

In this review, we distinguish between *representations*, which refer to how scanpaths are encoded or transformed into alternative forms—*e.g.*, geometric trajectories, symbolic strings, attention maps—and *metrics*, which define quantitative functions operating on these representations to summarize, compare, or characterize gaze behavior. Our goal is not to provide an exhaustive technical treatment of each approach, but rather to propose a unified conceptual framework that organizes the diversity of existing methods and clarifies their assumptions, required inputs, and interpretability, along with references to formal mathematical descriptions and implementation details. Importantly, this article does not address *areas of interest* (AoIs), which fall outside the scope of the present review and are treated in a separate dedicated work. As will become apparent, several methods developed for scanpath analysis are conceptually related to AoI-based approaches, yet the symbolic nature of AoI representations warrants an independent treatment.

## 2 Single scanpath representation

In this section, scanpaths are analyzed independently by examining the sequential and spatial properties of fixation sequences. We focus on methods designed to characterize the structure of a single gaze trajectory, without explicit comparison across observers or trials. We first introduce foundational geometrical *metrics*, which operate directly on fixation coordinates to quantify the spatial extent, dispersion, and complexity of scanpaths.

Beyond such low-level descriptors, a large body of work relies on higher-level *representations* that transform scanpaths into alternative forms in order to emphasize specific dimensions of gaze behavior. These include spatial density and attention maps, which support intuitive visual inspection and lie at the intersection of eye-tracking research and visual analytics, as well as recurrence-based representations that highlight the temporal organization and self-similarity of gaze sequences. We also review symbolic string encodings, which discretize scanpaths into categorical sequences and form the basis of many sequence-analysis techniques.

For each family of methods, we discuss their underlying assumptions, typical parameterizations, interpretability, and main limitations, with particular attention to sensitivity to discretization, spatial resolution, and temporal binning. The metrics and algorithms discussed in this section are systematically summarized in Table 1, which specifies the required inputs, typical outputs, and key references for implementation.

**TABLE 1:** Single scanpath metrics and their required input representations.

Feature name	Input	Description	References
Length	Fixation sequence	Computes the total distance traveled by the gaze between successive fixation centroids	Goldberg and Kotval (1998)
Dispersion	Fixation coordinates	Computes the standard deviation of fixation coordinates within a scanpath	Guo et al. (2023)
Successive angles	Fixation sequence	Computes the angles formed by successive saccadic trajectories between fixations	Goldberg and Kotval (1998)
Spatial density	Fixation coordinates	Computes the proportion of the visual field foveated during a task using circular filters centered on fixations	Castelhano et al. (2009)
K-coefficient	Fixation durations + saccade amplitudes	Computes, for each fixation, the difference between standardized fixation duration and standardized amplitude of the subsequent saccade	Krejtz et al. (2016)
Nearest neighbor index	Fixation coordinates	Computes the mean minimum inter-fixation distance normalized by the expected value under spatial randomness	Di Nocera et al. (2006)
Voronoi cells	Fixation coordinates	Computes statistical parameters — *e.g.*, skewness, scale — of a gamma distribution fitted to normalized Voronoi cell areas	Over et al. (2006)
Convex hull	Fixation coordinates	Computes the area of the smallest convex polygon containing all fixation points of a scanpath	Bhattacharya et al. (2020)
Higuchi fractal dimension	Fixation sequence (Hilbert-transformed)	Computes the Higuchi fractal dimension of the one-dimensional Hilbert-curve distance series derived from fixation centroids	Newport et al. (2021)
Saliency map	Fixation coordinates	Computes a fixation density map using Gaussian kernel smoothing over fixation locations	Bojko (2009)
Saliency map entropy	Saliency map	Computes the Shannon entropy of the normalized attention map distribution	Gu et al. (2021)
RQA recurrence rate	Fixation sequence	Computes the percentage of recurrence points in the recurrence matrix	Webber and Zbilut (1994)
RQA determinism	Fixation sequence	Computes the percentage of recurrence points forming diagonal line structures	Webber and Zbilut (1994)
RQA laminarity	Fixation sequence	Computes the percentage of recurrence points forming vertical or horizontal line structures	Webber and Zbilut (1994)
RQA CORM	Fixation sequence	Computes the distance between the center of recurrence mass and the main diagonal of the recurrence plot	Anderson et al. (2013)
RQA entropy	Fixation sequence	Computes the Shannon entropy of the diagonal-line length distribution in the recurrence plot	Marwan et al. (2007)

### 2.1 Geometrical approaches

From the earliest studies of eye movement behavior in observational tasks (Buswell, 1935), it was recognized that simple descriptive and geometric characterizations of scanpath trajectories could offer valuable insights into the underlying cognitive processes. With this in mind, we begin our overview by introducing several intuitive metrics that capture the spatial and geometric features of gaze trajectories.

#### 2.1.1 Basic descriptive features

A frequently studied feature in the literature is the *scanpath length*, which quantifies the total distance traveled by the eye during scanning. This metric is typically expressed in degrees of visual angle or pixels. To ensure meaningful interpretation, *scanpath length* is often normalized by time or analyzed within the framework of specific tasks or sub-tasks. High values of *scanpath length* are often associated with less efficient search behavior, as they reflect extensive eye movement without rapidly converging toward task-relevant information (Goldberg and Kotval, 1998). This metric has proven useful in various contexts. For instance, it has been employed to assess the diagnostic skills of medical students, pathology residents, and practicing pathologists when analyzing histopathology slides, revealing differences in scanning strategies and expertise (Krupinski et al., 2006). In clinical research, scanpath length has also been interpreted to characterize restricted scanning behaviors. For example, it has highlighted the limited exploration strategies observed in patients with schizophrenia, providing insights into their oculomotor dysfunction (Toh et al., 2011).

In addition to scanpath length, another valuable approach involves analyzing the angles formed by successive fixations along the scanpath trajectory. These angles are calculated based on two consecutive line segments connecting three fixations—previous, current, and next. They provide a way to characterize the geometric efficiency of visual search, with smaller and more direct angles often indicative of more focused behavior (Goldberg and Kotval, 1998). The analysis of angular distributions within scanpaths can be conducted independently or in combination with advanced modeling techniques. For example, Mao et al. (2022) used angular distributions to quantify task performance, while Fuhl et al. (2019) proposed leveraging sequences of saccadic angles for scanpath comparison. Similarly, Kümmerer et al. (2022) utilized inter-fixation angles as a validation metric for computational models of human scanpaths, demonstrating their relevance for benchmarking algorithms designed to replicate human visual behavior.

Another widely used descriptor is *fixation dispersion*, also known as spread, which assesses the spatial distribution of fixations. Dispersion can be computed in various ways, such as by calculating the standard deviation of fixation coordinates across a scene (Guo et al., 2023; Ryerson et al., 2021) or by measuring the deviation from a central reference point, often referred to as *dispersion from the center* (Anliker et al., 1976). This measure offers valuable insights into spatial viewing strategies and has been applied, for instance, to differentiate visual search strategies between novice and expert pathologists (Jaarsma et al., 2014). High fixation dispersion may reflect exploratory search patterns, whereas low dispersion can indicate focused attention—or, in some clinical or atypical populations, restricted exploration that is not necessarily efficient. This underlines the importance of interpreting these metrics in the context of the task, stimulus, and population under study.

Finally, many studies complement global scanpath metrics with descriptive measures of individual fixational and saccadic components. Examples include the mean *saccade amplitude* and the mean *fixation duration*. These measures help provide a more detailed characterization of oculomotor behavior and are particularly useful for comparing performance across tasks or populations. For a more comprehensive treatment of these descriptors, readers are referred to the *Oculomotor Processing* part of this review series (Laborde et al., 2025a), where the features used to characterize canonical oculomotor events are examined in detail.

Fundamental scanpath metrics such as *scanpath length*, angular analysis, and *fixation dispersion* provide complementary insights into the global structure of visual exploration. They are particularly appropriate in tasks where overall search efficiency, spatial spread, or exploratory style is of interest, such as visual search, inspection, and reading. When complemented by detailed measures of individual fixations and saccades, these metrics enable a more nuanced and comprehensive understanding of oculomotor behavior across a wide range of experimental and clinical contexts.

#### 2.1.2 Spatial density

A prominent global search metric, introduced by Kotval and Goldberg (1998), is the *scanpath spatial density*. This descriptive measure, computed independently of the temporal order of fixations, characterizes how widely the visual field is explored. A broadly distributed pattern of fixations typically reflects extensive searching, whereas fixations concentrated within a limited region suggest a more direct or focused exploration strategy. Consequently, spatial density has been employed to assess viewer expertise during complex cognitive tasks, with higher density often linked to more systematic and skillful performance (Augustyniak and Tadeusiewicz, 2006). Alternatively, spatial density can also be interpreted as a measure of scanpath regularity, which is particularly relevant in reading and comprehension studies (Mézière et al., 2023; von der Malsburg et al., 2015).

From a computational perspective, the earliest method for estimating spatial density relied on superimposing a regular grid over the visual field (Goldberg and Kotval, 1998). Fixations are mapped onto the grid, and the density is defined as the proportion of grid cells containing at least one fixation relative to the total number of cells. While straightforward, this approach is limited by the arbitrary choice of grid resolution, which directly influences the resulting density estimate. To alleviate this dependency, Castelhano et al. (2009) proposed a continuous alternative that avoids grid-based discretization. In this method, the proportion of the visual field foveated during a search task is computed by centering a circular filter—typically with a radius of 1° or 2° of visual angle—on each fixation. The union of the covered areas, normalized by the total visual field area, provides a smoother and more physiologically grounded density estimate.

Recently, Krejtz et al. (2016) and Krejtz et al. (2017) introduced the *K coefficient* as an extension of the *saccade-fixation ratio*. Developed to explore the dynamics of visual scanning in tasks such as artwork and map viewing, this metric averages the differences, for each fixation, between the standardized *fixation duration* and the standardized *saccade amplitude* of the subsequent saccade. The *K coefficient* has proven effective in distinguishing between ambient and focal attention states and serves as an indicator of cognitive load changes. Its ability to capture subtle shifts in attention dynamics makes it an effective tool for both experimental and applied research.

Another innovative metric, the *nearest neighbor index* (NNI), evaluates the randomness of fixation distribution across the visual field (Di Nocera et al., 2006). The NNI is computed as the mean of the minimum distances between fixation points, normalized by the expected mean distance under a random distribution. This metric has proven useful in assessing the relationship between fixation patterns and cognitive workload. For instance, lower workload conditions often correspond to more regular fixation distributions, suggesting systematic monitoring of an interface or visual layout.

**FIGURE 2:**
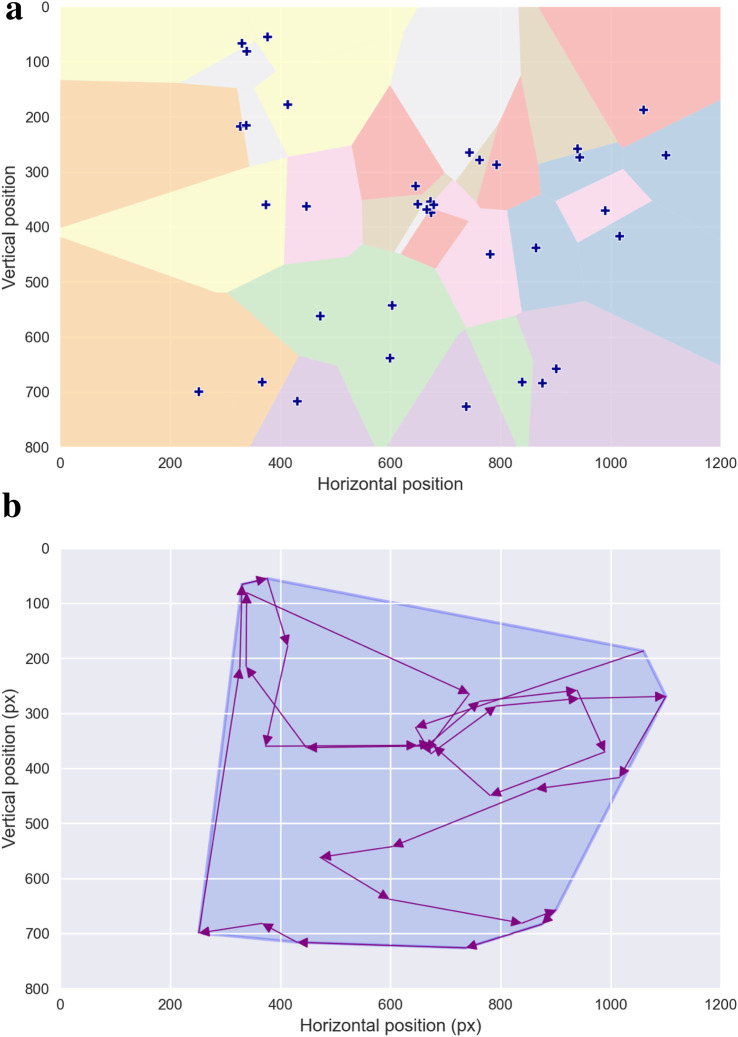
Geometrical Analysis. **(a)** illustrates the Voronoi tessellation derived from the scanpath shown in Figure 1. Each fixation serves as a generator point, defining a corresponding Voronoi cell whose area reflects the local spatial density of neighboring fixations. **(b)** depicts the convex hull of the same scanpath, shown in light blue. The convex hull corresponds to the smallest convex polygon—defined by interior angles not exceeding 180°—that encloses the entire set of fixation locations, thereby providing a global measure of the spatial extent of visual exploration.

A more sophisticated density measure, introduced by Over et al. (2006), utilizes *Voronoi diagrams* to characterize fixation uniformity. This method assigns each fixation a unique region of the visual field, known as a Voronoi cell, which comprises all points closer to that fixation than to any other—an illustration is provided in Figure 2a. The size and shape of these cells depend on factors such as the visual stimulus characteristics, the total number of fixations, and their spatial arrangement. This approach enables detailed analysis of fixation density by extracting descriptors from the distribution of Voronoi cell sizes, such as skewness or parameters of a gamma distribution. These descriptors provide insights into the uniformity and clustering of fixations, offering a powerful tool for understanding how visual attention is distributed during cognitive processes.

Overall, spatial density approaches are particularly well suited for research questions concerned with how *thoroughly*, *widely*, or *uniformly* a stimulus is explored, or for distinguishing between ambient and focal viewing modes, rather than for capturing the precise temporal ordering of fixations.

#### 2.1.3 Convex hull

The concept of the *convex hull* of fixations was introduced early on as a natural extension to the scanpath length metric (Kotval and Goldberg, 1998). The convex hull is defined as the smallest convex polygon encompassing all fixation points for a given participant under a specific experimental condition. This can be visualized as the area bounded by a tightened rubber band stretched around all fixation points until it encloses them completely—see Figure 2b for an illustration. The convex hull area provides an estimate of the extent of the peripheral visual field explored during a task (Bhattacharya et al., 2020). This metric has been widely employed to assess visual effort and attention distribution across various tasks and experimental conditions (Fu et al., 2017; Goldberg and Kotval, 1999; Imants and de Greef, 2011; Moacdieh and Sarter, 2015; Sharafi et al., 2015a). A consistent observation in these studies is that smaller convex hull areas correspond to more concentrated fixations and reduced visual effort, often indicative of a task-focused approach. For this reason, convex hull area is frequently analyzed in conjunction with scanpath length, as the two metrics together offer complementary insights into the spatial extent and efficiency of visual search.

While the convex hull area measure is a useful metric, it has significant limitations. A key drawback is its sensitivity to outliers and stray fixations, which can significantly distort the results. For instance, as noted by Bhattacharya et al. (2020), a scanpath with a few stray fixations near the corners of a region may produce a convex hull area comparable to that of a scanpath reflecting concentrated, systematic exploration of the same region. This highlights the challenge of using convex hull area in isolation, as it may fail to distinguish between meaningful search patterns and scattered fixations unrelated to the task—outlier fixations, even if rare, can disproportionately expand the convex hull and distort results (Sharafi et al., 2015a; b). Moreover, as an aggregated metric computed after a visual search sequence, its relevance can vary depending on the specific visual task, sometimes leading to misinterpretations.

To address these limitations, researchers have developed refined convex hull-based measures that incorporate temporal and fixation-density dimensions. Notably, Bhattacharya et al. (2020) introduced two refined metrics to enhance the analysis of visual search behavior: the *hull area per time*, which combines the dynamic convex hull area with the elapsed task duration to provide a time-normalized measure of the search spread, and the *fixations per hull area*, which integrates the running count of fixations with the corresponding convex hull area, offering a quantitative indicator of fixation density within the explored region. These enhanced features aim to provide more nuanced insights into visual behavior by addressing the static and outlier-sensitive nature of the raw convex hull area. Convex-hull-based metrics are therefore best used as global indicators of spatial extent or visual effort, and ideally in combination with other measures that capture fixation density or temporal dynamics.

#### 2.1.4 Fractal dimension

The concept of *fractal dimension* can be intuitively explained using the classic problem of measuring the coastline of an island. As the scale of measurement becomes smaller, the length of the coastline increases, making it increasingly difficult to measure accurately at finer scales, such as the granularity of a single grain of sand. This phenomenon highlights the complexity of irregular structures, and to quantify such complexity, a powerful tool was introduced: the *box-counting dimension*, also known as the Minkowski–Bouligand dimension. To compute the *box-counting dimension*, the fractal structure is overlaid with a grid of evenly spaced boxes. The number of boxes required to cover the structure is then counted, and the dimension is determined by observing how this count changes as the size of the grid cells is reduced. This approach is useful for quantifying the degree of irregularity in structures that exhibit fractal properties, which are often self-similar across scales.

Interestingly, the scanpath formed by connecting successive eye fixations during scene viewing or visual search tasks can be treated as a fractal pattern. Fractals are particularly effective at capturing spatial structures and offer valuable insights into the geometric organization or generation of scanpaths during cognitive tasks such as visual search or scene exploration (Cote et al., 2011). The *fractal dimension* has been employed to characterize human visual search behavior in diverse contexts, including mammography screening (Alamudun et al., 2017; 2015) and the analysis of brain magnetic resonance imaging (MRI) scans (Suman et al., 2021), as well as to explore its relationship with task complexity and reader expertise—for instance Wu et al. (2014) demonstrated the utility of this metric in quantifying scene complexity.

Traditional box-counting methods applied to the two-dimensional shape of scanpaths do not account for the temporal aspect of these eye movements. To address this limitation, Newport et al. (2021) recently introduced an alternative method that captures the fractal complexity of two-dimensional gaze patterns while incorporating the temporal dimension. Their method utilizes the *Higuchi fractal dimension* (HFD), an approximation of the Minkowski–Bouligand method specifically designed for one-dimensional time series. The primary advantage of HFD lies in its ability to directly analyze non-periodic, non-stationary data, which is characteristic of eye movement patterns.

**FIGURE 3:**
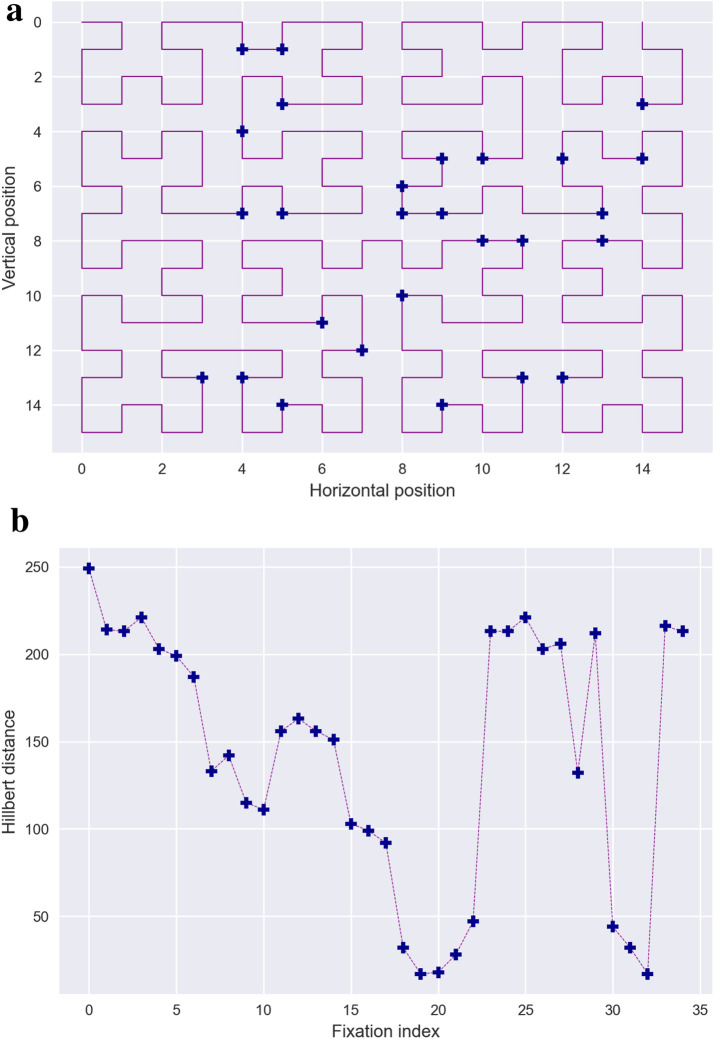
Higuchi Fractal dimension. **(a)** illustrates dimensionality reduction using the Hilbert curve. Fixations forming the scanpath are mapped onto a Hilbert curve, a space-filling curve that traverses the entire visual field. In this representation, Cartesian fixation coordinates are reduced to a single-dimensional coordinate representing their position along the Hilbert curve, starting from the origin at the bottom-left corner of the visual field. **(b)** plots the Hilbert curve distances against their temporal indices. Subsequently, the Higuchi method can be applied to estimate fractal dimensions. Briefly, this approach computes the lengths L(k) of sub-series extracted from the Hilbert distance series for various lags k between consecutive samples. Assuming a power-law relationship, $L\left(k\right)\propto k^{-D}$ , the fractal dimension D is estimated using logarithmic regression, as illustrated in Figure (c) .

Since the HFD method is applied to one-dimensional time series, ithe two-dimensional positional data of scanpaths must first be transformed into a single one-dimensional sequence. Newport and colleagues addressed this dimensionality reduction by employing Hilbert curve distances (Bially, 1969), a technique that maps two-dimensional scanpath coordinates into a one-dimensional sequence while preserving the spatial order of fixations. This transformation enables the application of the HFD method to characterize the fractal complexity of scanpaths, as illustrated in Figure 3. This two-step approach has proven particularly effective in filtering out outlier scanpaths that exhibit inconsistent or meaningless patterns, thereby enhancing the robustness of scanpath analyses (Newport et al., 2021; 2022). Fractal-based measures are therefore particularly appropriate when the research focus lies on the *complexity*, *irregularity*, or *self-similar structure* of exploration patterns, rather than on precise fixation locations or exact temporal ordering.

### 2.2 Saliency maps

The term *saliency map* can be a source of confusion due to its broad application across various research domains, where it encompasses different conceptualizations and uses. It has been described in multiple, overlapping contexts: as an abstract map for attentional priority, as a neural mechanism for integrating visual activity, as a bottom-up predictor of gaze locations, and as any heatmap-like representation of fixation series (Foulsham, 2019). In the following sections, we focus on two specific interpretations of saliency maps. First, we introduce *attention maps*, or *heat maps*, which are commonly used techniques for visualizing gaze data and naturally extend the concept of scanpath density. Second, we provide an overview of *saliency models*, which generate maps that estimate the likelihood of different image regions attracting an observer’s attention. These models are typically grounded in computational neuroscience and computer vision, aiming to predict the areas where visual attention is most likely to be directed based on image characteristics.

#### 2.2.1 Attention maps

**FIGURE 4:**
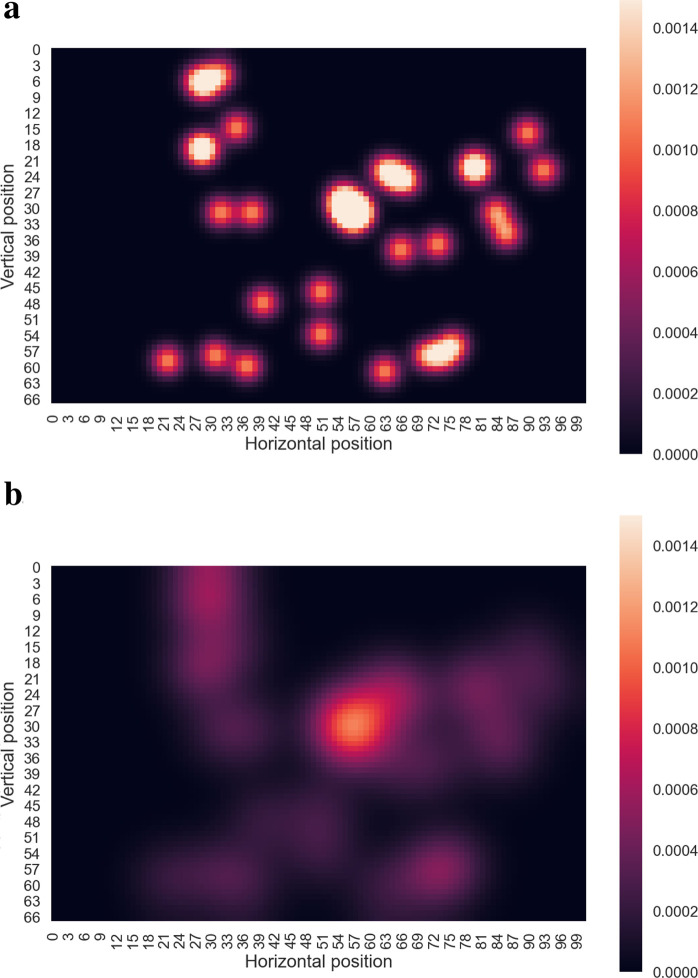
Attention Maps. Two attention maps derived from the same scanpath illustrated in Figure 1b. **(a,b)** specifically illustrate attention maps generated using Gaussian kernels with low and high standard deviation values, respectively. These examples highlight the significant influence of the Gaussian dispersion parameter, which must be carefully calibrated to accurately represent the variability and resolution of the visual system. Note that attention maps are computed on a user-defined grid whose resolution is independent of the original stimulus. As a result, the coordinate axes in these maps differ from those in Figure 1b.

A viewer’s *attention map*—often referred to as a *heat map*—is a widely used visualization of the spatial distribution of visual fixations across a stimulus. Conceptually, attention maps are spatial density plots that indicate how frequently different regions of the visual field are inspected. They can be understood as a continuous analogue of a histogram, where fixations, from a single observer or aggregated across observers, are accumulated on a discretized grid, and the fixation counts determine the resulting pixel intensities—typically indicated by color gradients or opacity. Importantly, the resolution of this grid is *chosen by the user* and does not necessarily match the original resolution of the stimulus; it is a modelling choice that influences the smoothness and spatial precision of the map. To generate a continuous density field, each fixation is typically convolved with a Gaussian kernel whose standard deviation determines how broadly the fixation spreads across the visual field. The choice of this parameter is critical, as it should reflect eye-position uncertainty and foveal extent, and is often set to 1 or 2 degrees of visual angle. As illustrated in Figure 4, varying the Gaussian dispersion parameter directly affects the granularity and interpretability of the resulting attention map.

This general description must be nuanced by several important considerations. While the *fixation-count* attention map, which aggregates the number of fixations, is an intuitive and straightforward representation, it has inherent limitations that can affect its interpretability and reliability. Most notably, this method assigns equal weight to all fixations, irrespective of their duration. Consequently, regions with similar intensity on a fixation-count map do not necessarily correspond to equivalent total gaze durations. For example, a brief glance repeated several times in one area may be indistinguishable from prolonged sustained attention in another, despite the potentially different cognitive or perceptual implications of these gaze patterns.

Furthermore, when fixation-count maps are generated from data collected across multiple observers, they can inadvertently introduce biases. For instance, observers who are exposed to the stimulus for longer durations naturally have more opportunities to produce fixations, disproportionately influencing the overall map. This effect can skew the representation toward their individual viewing behavior, especially in datasets where exposure times vary significantly among participants. It is also important to note that the idiosyncratic interests of certain observers can introduce bias. Individuals with particularly high interest in specific items or regions may contribute a disproportionately large number of fixations to those areas, overshadowing the collective patterns of the broader group. As a result, fixation-count maps may over-represent such idiosyncrasies, reducing their ability to generalize about attention allocation across a population.

To mitigate these shortcomings, alternative methods have been proposed that incorporate additional dimensions of visual behavior (Bojko, 2009). One such approach is the *absolute gaze duration* attention map, which represents the total time observers spend fixating on different areas of a stimulus. This method highlights regions that consistently attract sustained attention, offering insights into areas of prolonged engagement. However, it may still be influenced by differences in exposure time among observers or individual variability in attention patterns, potentially introducing bias into the results.

Another approach is the *relative gaze duration* attention map, which normalizes gaze duration data by calculating the time spent fixating on each area as a proportion of the total viewing time for each observer. This normalization reduces biases caused by variations in individual exposure times or personal viewing tendencies, enabling more equitable comparisons across participants. Despite its advantages, this method may obscure absolute differences in gaze duration between regions or participants, which could be significant for certain analyses.

A third method is the *participant-percentage* attention map, which reflects the proportion of observers who fixate on specific areas of a stimulus. This approach is particularly useful for identifying regions that consistently attract attention in a population and highlighting universally salient or compelling features. However, since it does not account for the frequency or duration of fixations, it is less effective in assessing the intensity or depth of attention directed toward specific areas.

Each of these methods has unique strengths and weaknesses, and their suitability depends on the research objectives and the experimental paradigm. For example, absolute or relative gaze-duration maps are often preferred in studies focusing on sustained attention, while participant-percentage maps are more appropriate for understanding population-wide trends in visual salience. For further discussion on this conceptual topic, we refer the reader to Bojko (2009), who provide guidelines for avoiding the misuse and misinterpretation of attention maps. They stress that attention maps, regardless of the method used to create them, must be interpreted carefully, as the choices made during their construction can significantly influence the conclusions drawn from the data. By aligning methodological choices with the specific aims of a study, researchers can maximize the accuracy and relevance of their findings.

Owing to their simplicity, intuitive readability, and strong visual appeal, attention maps have become a widely adopted tool for illustrating what captures viewers’ gaze. They offer a qualitative representation of attentional allocation and are employed across numerous domains. In marketing, they are used to analyze consumer focus, inform strategies for product placement, and optimize the visual layout of advertisements and interfaces (Li et al., 2016; Pan et al., 2011). In ergonomics, they guide the design of more efficient workplace layouts and support usability improvements in human–machine interaction (Bhoir et al., 2015). In psycholinguistics, attention maps contribute to the study of reading patterns and the cognitive mechanisms underlying language comprehension (Liu and Yuizono, 2020). In cognitive assessment, they provide insights into individual differences in perceptual and attentional processing, shedding light on both typical and atypical developmental trajectories (Pettersson et al., 2018). Beyond classical eye-tracking applications, attention maps can be seen as part of a broader *visual analytics* framework, in which interactive visualizations support exploration and interpretation of complex gaze data.

Conceptually, attention maps have long demonstrated that visual fixations are not uniformly distributed throughout the viewer’s field of vision. One key observation, noticed as early as the foundational studies of gaze behavior in complex scenes (Buswell, 1935), is the presence of a central bias, where fixations tend to cluster near the center of the visual field. This phenomenon has since been consistently confirmed in a variety of experimental paradigms (Mannan et al., 1995; 1996; 1997), reinforcing its robustness as a characteristic of gaze distribution.

Attention maps, however, offer a *static* visualization of averaged spatial scanpaths, providing no direct information about the temporal dynamics of gaze behavior, such as the sequence or duration of fixations. Additionally, while attention maps approximate the spatial distribution of visual attention, they remain largely qualitative in nature. Attempts to quantify these distributions, such as using metrics like *heatmap entropy* (Gu et al., 2021), remain relatively rare. Quantitative analyses typically necessitate comparative approaches, as outlined in Sections 3.3.1, 3.3.2, emphasizing the importance of robust methodological frameworks for interpreting attention maps. In practice, attention maps are most useful as intuitive visual summaries or as components of visual analytics pipelines, often combined with scanpaths or other representations.

#### 2.2.2 Saliency models

Similar to attention maps, *saliency models* are concerned with spatial distributions of attention, but they refer specifically to computational frameworks designed to *predict* the regions of an image or scene where individuals are most likely to focus their visual attention. Rooted in the concept of visuo-spatial attention, these models aim to explain how humans allocate attention to areas perceived as most salient or important. While the detailed development of saliency models falls outside the scope of this review, which focuses on eye-tracking data analysis, we briefly outline key aspects of these models and their applications across diverse domains.

One central function of the human visual system is to direct attention toward regions of the visual environment that are perceived as salient—areas likely to contain important information or require further cognitive processing. Evidence suggests that specific brain regions, particularly those in the frontal and parietal lobes responsible for controlling eye movements, may act as a *saliency map* (Treue, 2003). These regions are thought to encode spatial priorities, integrating bottom-up sensory inputs with top-down cognitive factors such as intentions, expectations, and goals (Bisley and Goldberg, 2010; Zelinsky and Bisley, 2015). The *biased competition theory* of attention (Maunsell and Treue, 2006; Beck and Kastner, 2009; Schoenfeld et al., 2014) provides a robust framework for understanding this process. According to the theory, bottom-up visual features—such as color, contrast, and motion—compete for attentional resources but are dynamically influenced by top-down factors like task goals or expectations. This interaction results in a competitive process where stimuli that are most relevant or task-critical ultimately *win*, directing cognitive and perceptual focus to areas of highest priority.

From a computational perspective, early saliency models, such as the influential framework proposed by Koch and Ullman (1985), introduced the concept of modeling visual attention as a topographical salience map. In this approach, regions of the visual field more likely to attract attention are assigned higher saliency values, producing a two-dimensional map that encodes the relative prominence of various areas. The allocation of attention is then governed by a *winner-takes-all* mechanism, in which the most significant region is prioritized as the target for the next fixation. The saliency at each location reflects its capacity to draw attention, with higher values indicating an increased likelihood of directing visual processing to that area.

Building upon this foundational concept, Itti and Koch (2000) developed a more sophisticated computational model that incorporated a range of low-level visual features, such as color, intensity, orientation, and contrast. This model used a parallel processing architecture where each feature was processed through separate channels, with each channel contributing to the overall saliency map. By integrating these diverse features, their model generated a saliency map that more accurately reflected the complex, multidimensional nature of visual attention. Specifically, the saliency value of each pixel was determined by combining the outputs of the different feature channels.

Over the years, the field of saliency modeling has matured significantly, with numerous new models being published regularly, each introducing new features and improvements. Many of these models focus on detecting visually interesting regions of an image, with applications in areas such as automated object detection, autonomous vehicle navigation, and real-time video compression. The original Itti-Koch model has been refined over time to include additional features like log spectrum (Hou and Zhang, 2007), entropy (Wang et al., 2010), histograms of oriented gradients (Ehinger et al., 2009), and center bias (Tatler, 2007), all of which help to better approximate human visual attention. Recently, models have also begun incorporating top-down modulation, allowing them to account for context or task-specific priorities in guiding attention.

The success of deep learning approaches has further revolutionized the field. Today, fully convolutional neural networks (CNNs) dominate the landscape of saliency models, offering improved performance through the use of large-scale datasets and powerful feature-learning algorithms (Wang et al., 2021). These deep saliency models have significantly advanced the accuracy of predicting where people will look in complex scenes, marking a new era in the study of visual attention. The topic of predicting human scanpaths when viewing visual stimuli lies beyond the scope of this work. For further information on this subject, we refer the reader to recent studies, including Kümmerer and Bethge (2021), Yang et al. (2024), Sui et al. (2023), and Li et al. (2024). In the context of this review, saliency models are primarily relevant as generators of predicted attention maps that can be compared with empirical scanpath-based representations.

### 2.3 Recurrence quantification analysis

The methods introduced so far have focused primarily on the spatial structure of scanpaths. However, many aspects of gaze behavior—such as repeated inspections of the same region, the ordering of fixations, or the persistence of specific scanning routines—are inherently temporal. Capturing these temporal properties requires a different analytical strategy. *Recurrence quantification analysis* (RQA), originally developed to study nonlinear and dynamical systems (Eckmann, 1987; Webber and Zbilut, 1994), provides such a framework and has proven particularly effective for analyzing the temporal evolution of eye movements.

RQA provides a versatile framework for quantifying the temporal organisation of fixation sequences, offering metrics that describe how often—and in what manner—a scanpath revisits previously observed states. In the context of gaze behaviour, these *states* correspond to fixation locations, and RQA metrics capture temporal regularities such as re-inspections, repeated subsequences, or periods of sustained attention within a given region. The first formal application of RQA to scanpath analysis was introduced by Anderson et al. (2013), who demonstrated that recurrence-based measures reveal meaningful temporal structure across observers and tasks. Their pioneering work has since inspired a broad range of studies showing that RQA-derived measures are sensitive to variations in scene complexity and visual clutter (Wu et al., 2014), as well as to differences in expertise, cognitive load, and attentional strategy (Vaidyanathan et al., 2014; Farnand et al., 2016; Gandomkar et al., 2018; Perez et al., 2018; Gurtner et al., 2019). Collectively, these findings illustrate how RQA complements spatial metrics by emphasizing the dynamic unfolding of fixations over time, thereby enriching our understanding of gaze behaviour and its relation to visual and cognitive processing.

#### 2.3.1 Towards a recurrence plot

To fully comprehend this approach, it is crucial to first understand the concept of *recurrence plots*. These plots, fundamental to recurrence quantification analysis (RQA) methodologies (Eckmann et al., 1987), visually represent the recurrent patterns of fixations. Introducing recurrence plots establishes the foundation for analyzing their role in interpreting scanpath dynamics.

**FIGURE 5:**
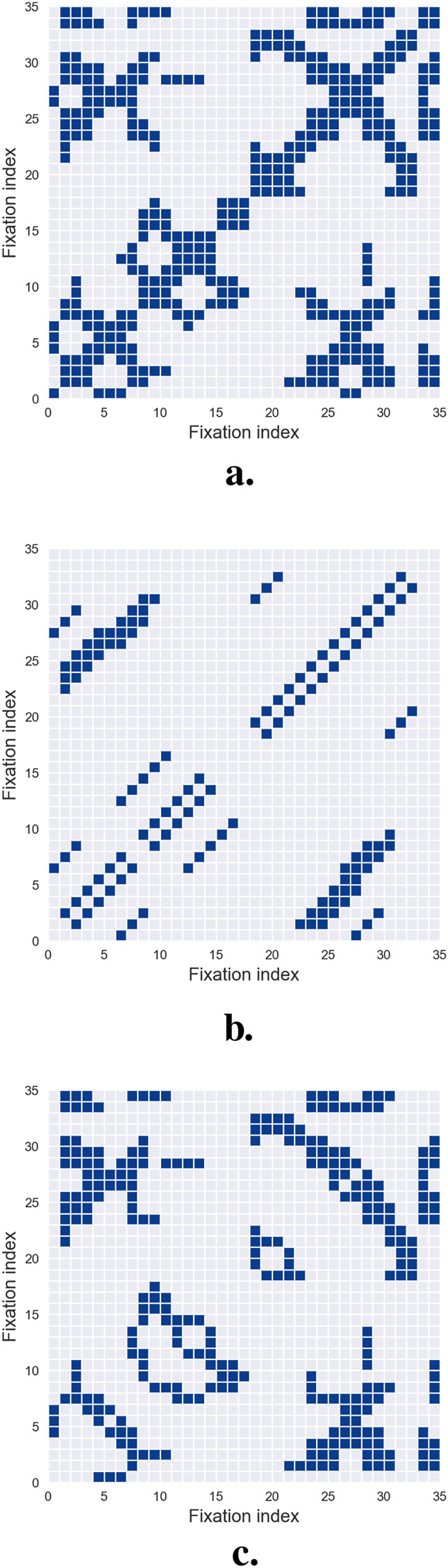
Recurrence Quantification Analysis. **(a)** illustrates a recurrence plot, where the columns and rows correspond to the fixations of the analyzed scanpath. A dot is placed at position (i,j) if the i -th fixation is sufficiently close to the j -th fixation, indicating spatial recurrence. **(b)** highlights all diagonal lines of at least three points extracted from the recurrence plot, which represent repeated patterns and are used to calculate *determinism*. **(c)** depicts the horizontal and vertical lines extracted from the recurrence plot, representing re-scanning sequences, which are used to compute *laminarity*.

A recurrence plot is a square array constructed from a scanpath, where a dot is placed at the (i,j) -th entry whenever the i -th fixation is sufficiently close to the j -th fixation. Each dot, referred to as a recurrence point, indicates that the scanpath trajectory has returned to a previously visited location, within a small error tolerance. As illustrated in Figure 5a, the recurrence plot visualizes the set of all pairs of time indices where such recurrences occur. Conceptually, it corresponds to a square recurrence matrix where each element represents the proximity of two fixations within a predefined cutoff limit. Typically, recurrence points are binary, with the (i,j) -th entry assigned a value of 1 to signify recurrence. However, some studies propose incorporating temporal weighting by adjusting the value of each recurrence point based on the combined durations of the i -th and j -th fixations in the scanpath, adding a temporal dimension to the analysis.

One significant challenge in (RQA) is selecting an appropriate distance threshold to define recurrence. If the threshold is set too low, the recurrence plot may display few or no recurrence points, rendering the analysis uninformative. Conversely, an overly high threshold results in excessive recurrences, where nearly all points are neighbors, obscuring meaningful patterns. Currently, no universal threshold is applicable across all experimental paradigms. Instead, the threshold must be carefully calibrated based on context-specific rules and heuristics (Zbilut et al., 2002), with particular attention to the semantic density of the visual field being analyzed.

Recurrence plots are inherently symmetrical about the main diagonal, allowing all relevant information to be extracted from the upper triangle while excluding the main diagonal and lower triangle. Upon qualitative examination, recurrence plots often reveal distinct short line segments parallel to the main diagonal, representing clusters of fixations associated with brief periods of consistent gaze behavior. Additionally, isolated points may appear, reflecting sporadic or chance recurrences.

To move beyond qualitative visual inspection, researchers have developed systematic methods for extracting quantitative characteristics and metrics from recurrence plots. These automated techniques enable detailed characterization of recurrence patterns, providing a more rigorous basis for analysis. The next section details these metrics and their application to scanpath studies.

#### 2.3.2 Recurrence quantitative features

Once a recurrence plot has been constructed, several quantitative measures can be derived to characterize how a scanpath unfolds over time. The most direct of these is the *recurrence rate*, defined as the percentage of fixation pairs that fall within the recurrence threshold. This descriptor—introduced to scanpath analysis by Anderson et al. (2013) following earlier developments in nonlinear time-series analysis (Eckmann, 1987; Webber and Zbilut, 1994) — captures how often observers return to locations previously fixated during exploration.

A second feature, *determinism*, quantifies the percentage of recurrence points that align to form diagonal line segments in the plot, as shown in Figure 5b. These diagonals reflect the repetition of short subsequences of fixations and therefore index the predictability or stereotypy of gaze behavior. High determinism often emerges in tasks involving structured comparisons or repeated scanning routines, as illustrated in several applied studies (Vaidyanathan et al., 2014; Farnand et al., 2016; Perez et al., 2018). Complementary to this, *laminarity* measures the extent to which recurrence points form vertical or horizontal lines, as shown in Figure 5c. These features correspond to prolonged dwell times or repeated returns to specific regions, and have been shown to relate to task demands and the semantic structure of the stimulus (Anderson et al., 2013; Gandomkar et al., 2018; Gurtner et al., 2019).

A more global descriptor, the *center of recurrence mass* (CORM) reflects the temporal distribution of recurrent points. It is defined as the distance between the center of gravity of the recurrence points and the main diagonal of the recurrence plot—representing self-recurrence (Anderson et al., 2013). A small CORM value indicates that re-fixations are closely spaced in time, while a larger CORM suggests that re-fixations are more spread out. Together with the recurrence rate, CORM captures the global temporal structure of fixation sequences, while determinism and laminarity provide insights into local gaze patterns.

Finally, *entropy* characterizes the complexity of the recurrence structure by computing the Shannon entropy of the distribution of diagonal line lengths (Shannon, 1948; Lanata et al., 2020). Although less frequently reported in the gaze literature (Villamor and Rodrigo, 2017), entropy is informative about the diversity of repeated patterns: low values reflect highly regular or stereotyped behavior, whereas high entropy indicates more variable and irregular recurrence structures.

Together, these quantitative features provide a multidimensional characterization of the temporal organization of scanpaths, capturing tendencies toward repetition, revisits, temporal clustering, and structural complexity. They offer a principled way to summarize dynamic viewing behavior and have been successfully applied across a wide range of visual tasks and experimental domains. Several open-source toolboxes provide implementations of RQA and CRQA for eye-tracking and time-series data, including the *CRP Toolbox* for MATLAB (Marwan et al., 2007) and Python-based libraries such as *pyRQA* (Rawald et al., 2017), which facilitate reproducible and scalable applications of recurrence-based methods.

Beyond the characterization of a single scanpath, the same methodological principles extend naturally to the comparison of two observers or two viewing conditions. This approach, known as *cross-recurrence quantification analysis* (CRQA), replaces the self-comparison of a scanpath with a joint recurrence plot constructed from two separate gaze sequences. Whereas RQA identifies how an individual revisits locations over time, CRQA captures how two scanpaths converge, diverge, or realign as they evolve. This makes CRQA particularly suitable for studying inter-observer consistency, shared viewing strategies, or condition-dependent synchrony in gaze behavior. The specific metrics and methodological considerations associated with CRQA are detailed in Section 3.4, where we examine its role within the broader landscape of scanpath comparison techniques.

Although RQA and areas of interest (AoI) analysis may appear conceptually related—both seek to identify stable patterns and revisitations within a scanpath—their objectives and assumptions differ in important ways. AoI analysis relies on predefined, semantically meaningful regions of the stimulus, and focuses on how often, in what order, and for how long these regions are fixated. RQA, in contrast, operates without any semantic partitioning of the visual field: it quantifies recurrence directly from the geometry and temporal structure of the fixation sequence. As a result, RQA can reveal regularities, cycles, or temporal dependencies that extend beyond the boundaries of any *a priori* region definition. Conversely, AoI methods offer interpretability grounded in stimulus meaning, which RQA does not provide on its own. These approaches are therefore complementary rather than interchangeable. A fuller discussion of AoI techniques and their methodological implications is provided in a separate dedicated work.

### 2.4 String sequence representation

A notable way to represent scanpath trajectories relevant to this discussion is to transform them into *string sequences*. In this approach, the visual field is discretized by superimposing a static two-dimensional grid onto the stimulus, with each grid cell assigned a symbolic label, typically an alphabetic character. Each fixation is then mapped to the corresponding cell, transforming the spatial progression of gaze points into an ordered sequence of symbols. This symbolic encoding recasts the scanpath as a string, yielding a compact and structured representation that preserves the temporal order of visited regions while deliberately abstracting away fine-grained spatial detail.

From a qualitative standpoint, this representation is particularly advantageous because it suppresses low-level geometric variability while retaining the meaningful organization of the observer’s visual exploration. By reducing a continuous trajectory to a sequence of symbolic transitions, recurring patterns become easier to detect—such as preferred regions of interest, characteristic scanning strategies, or stimulus-driven exploration pathways. The resulting strings also lend themselves to intuitive comparisons across observers: similarities and differences in viewing patterns can often be perceived at a glance, without the need for detailed geometric analysis. In this way, string-based representations foreground the *qualitative structure* of visual behavior, making complex spatio-temporal dynamics more interpretable and more amenable to systematic comparison.

Furthermore, the string-sequence representation provides a foundational basis for a wide range of string-based scanpath comparison algorithms, which will be examined in subsequent sections, particularly in Sections 3.2, 3.5. These methods operate directly on the symbolic sequences to quantify similarities or differences between scanpaths, thereby enabling systematic comparisons across observers, stimuli, or experimental conditions.

While this approach facilitates the conversion of continuous gaze data into a discrete format, the process of spatial binning demands careful consideration (Anderson et al., 2015). A fixed grid resolution may inadequately capture fine-grained fixation details in high-interest areas if the grid is too coarse; conversely, a grid that is too fine may introduce unnecessary complexity in low-salience or uniform regions. For this reason, it is often advantageous to adapt the grid resolution to the underlying image content, ensuring that meaningful regions are represented with adequate precision.

In cases where the scene contains large, visually variable but semantically uninformative areas, grid-based discretization may fragment these regions excessively, making cognitive interpretation more difficult. A common alternative is therefore to assign symbolic representations to predefined *areas of interest* (AoIs) based on their distinct semantic or functional roles (Josephson and Holmes, 2002b; West et al., 2006). This strategy aligns the discretization process with the structure of the scene and the expected attentional targets of viewers. However, it requires careful analysis of the image content and the viewer’s attention patterns, necessitating the use of specialized methodologies, which will be explored in detail in a separate dedicated contribution.

Beyond spatially defined discretization methods, other strategies focus on the statistical distribution of fixations rather than their geometric layout. One such method is *percentile mapping*, in which elements of the scanpath are mapped to a discrete alphabet so that each symbol appears with approximately equal frequency (Kübler et al., 2014). This normalization compensates for spatial offsets that may arise between different recording sessions or observers, providing a more balanced representation across datasets. Compared with grid-based methods, percentile mapping can therefore reduce bias introduced by uneven fixation density, offering improved comparability across heterogeneous stimuli (Kübler et al., 2017). This technique resembles the discretization procedure used in the well-known *SAX* (Symbolic Aggregate approXimation) representation for time series data (Lin et al., 2007), where continuous values are transformed into discrete symbols to facilitate analysis.

**FIGURE 6:**
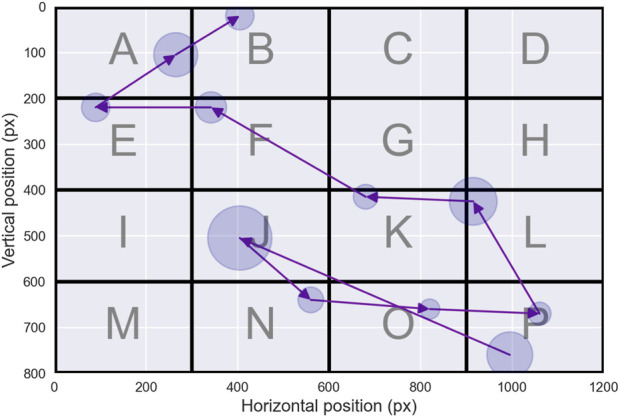
String Sequence. To convert a scanpath trajectory into a sequence of characters, the visual field is first divided into regions of equal size, each designated by a character, from A to P . Accordingly, each fixation is associated with a character to produce, based on the example trajectory illustrated above, the following sequence: PJNOPLKFEAB . Additionally, if a temporal binning is performed, each character is repeated in proportion to the corresponding fixation duration, to produce the following sequence: PPJJJJNOPLLKFEAAB .

One of the key challenges associated with converting scanpaths into string sequences is the loss of temporal information, particularly fixation duration, which is an integral component of eye movement behavior. To address this issue, it is possible to introduce temporal binning into the string sequence. This process involves repeating the symbol corresponding to a specific spatial region in proportion to the duration of the corresponding fixation (Cristino et al., 2010; Takeuchi and Matsuda, 2012). By encoding the fixation duration in this manner, the resulting string captures not only the spatial location and sequence of fixations but also the temporal dimension, offering a richer depiction of gaze behavior. In summary, the effectiveness of string-based representations critically depends on how spatial and temporal aspects of gaze are discretized and weighted in the resulting sequence. An example of this representation can be seen in Figure 6.

## 3 Similarity between scanpaths

As discussed earlier in this review, visual scanpaths are shaped by a combination of bottom-up and top-down factors, including the task assigned to viewers (Simola et al., 2008), the characteristics of the stimuli (Yarbus, 1967a), and individual variability (Viviani, 1990). Quantifying the differences or similarities between visual behaviors is therefore critical for understanding how these factors influence eye movements and for gaining deeper insights into the cognitive processes underlying visual attention.

Comparing visual scanpaths also plays a central role in *scanpath theory*. While early studies by Noton and Stark (1971a) and Noton and Stark (1971b) relied on visual inspection to evaluate scanpath similarity, the development of automated metrics began approximately two decades later (Brandt and Stark, 1997). Since then, the growing interest in analyzing eye movement sequences has led to the creation of numerous methodologies for the automated comparison of scanpaths. These methods differ in the representations they operate on—raw fixations, vectors, strings, saliency maps—in the aspects of behavior they emphasize—spatial overlap, temporal structure, pattern repetition—and in their computational demands. The comparison methods presented in this section are summarized in Table 2, which provides a concise description of each approach, the required input formats, and references from the literature that offer guidance for their implementation.

**TABLE 2:** Scanpath comparison methods and their required input representations.

Method name	Input	Description	References
Mannan distance	Fixation coordinates	Computes the weighted mean distance between each fixation in one scanpath and its nearest neighbor in the other — point-mapping	Mannan et al. (1995)
EyeAnalysis distance	Fixation coordinates + durations	Computes the sum of all point-mapping distances normalized by the number of points in the longer sequence	Mathôt et al. (2012)
TDE distance	Fixation sequences	Computes the time-delay embedding distance between two scanpaths	Wang et al. (2011)
DTW distance	Fixation sequences	Computes the temporal alignment that minimizes the Euclidean distance between aligned fixation points	Berndt and Clifford (1994)
Fréchet distance	Fixation sequences	Computes the minimum of the maximum distances between two scanpaths under continuous alignment with preserved ordering	Eiter and Mannila (1994)
Levenshtein distance	String sequences	Computes the minimum number of edits — insertions, deletions, substitutions — required to transform one scanpath into another	Wagner and Fischer (1974)
Generalized edit distance	String sequences	Computes the edit distance with distinct insertion, deletion, and substitution costs defined by a cost matrix	Wagner and Fischer (1974)
Needleman–Wunsch distance	String sequences	Computes an optimal global alignment with match bonuses and gap penalties using dynamic programming	Needleman and Wunsch (1970)
Normalized scanpath saliency	Fixations + saliency map	Computes a z-scored saliency value at fixation locations relative to the saliency map	Peters et al. (2005)
Saliency percentile	Fixations + saliency map	Computes the mean percentile rank of saliency values at fixation locations	Peters and Itti (2008)
Information gain	Fixations + saliency map	Computes the gain in predictive power of a saliency model relative to a center-prior baseline	Kümmerer et al. (2014)
Saliency AUC	Fixations + saliency map	Evaluates how well a saliency map predicts fixations using ROC curve analysis across thresholds	Bylinskii et al. (2018)
Kullback–Leibler divergence	Saliency maps	Computes the information loss when one saliency map approximates another	Le Meur et al. (2007)
Pearson correlation	Saliency maps	Computes the linear correlation coefficient between two saliency maps	Le Meur et al. (2006)
Earth mover distance	Saliency maps	Computes the minimum transport cost required to morph one saliency distribution into another	Riche et al. (2013)
CRQA recurrence rate	Fixation sequences	Computes the percentage of recurrent fixation pairs in the cross-recurrence matrix	Marwan et al. (2007)
CRQA determinism	Fixation sequences	Computes the percentage of cross-recurrent points forming diagonal line structures	Marwan et al. (2007)
CRQA laminarity	Fixation sequences	Computes the percentage of vertically aligned cross-recurrent points	Marwan et al. (2007)
CRQA entropy	Fixation sequences	Computes the Shannon entropy of the distribution of diagonal line lengths in the cross-recurrence plot	Marwan et al. (2007)
SubsMatch similarity	String sequences	Computes scanpath similarity from frequency differences of symbolic subsequences of size n	Kübler et al. (2014)
ScanMatch score	String sequences	Computes a similarity score using Needleman–Wunsch alignment with a spatial substitution matrix	Cristino et al. (2010)
MultiMatch alignment	Saccade vectors	Computes similarity across five dimensions: shape, length, position, direction, and fixation duration after vector alignment	Dewhurst et al. (2012)

### 3.1 Direct comparison

This first class of methods compares pairs of scanpaths directly in the spatial–temporal domain, without converting them into alternative symbolic or image-based representations. Such approaches preserve the original coordinate information and are particularly attractive when precise spatial relationships are important or when one wishes to avoid additional preprocessing steps such as discretization or spatial binning. We distinguish here simple point-mapping metrics from more sophisticated *elastic alignment* methods.

#### 3.1.1 Point mapping metrics

The Euclidean distance—also referred to as the *straight-line* distance—is one of the fundamental measures initially employed for comparing scanpaths. In its simplest form, this metric is calculated as the sum of the distances between corresponding fixations in two scanpaths. However, this naive approach was quickly deemed inadequate, as it implicitly assumes equal-length fixation sequences and strict one-to-one correspondence between fixations, a condition rarely met in practical applications.

To address this limitation, Mannan et al. (1995) introduced a seminal metric based on the weighted mean distance between each fixation in one scanpath and its nearest neighbor in the other—a technique often referred to as *point-mapping* (Mannan et al., 1995; 1996). Extending this principle, their double-mapping approach considers bidirectional mappings between two scanpaths and has inspired a broad family of metrics applicable to sequences of varying lengths. These methods have found utility in diverse research contexts, including studies on visual scanning behavior and scene perception (Pambakian et al., 2000; Foulsham and Underwood, 2008; Mannan et al., 2009; Shakespeare et al., 2015; Konstantopoulos, 2009).

Despite their utility, point-mapping techniques have notable limitations. A major drawback is their exclusive reliance on spatial properties, as they disregard the temporal order of fixations. Consequently, two scanpaths with reversed fixation sequences but identical spatial configurations will yield identical Mannan distances, ignoring the sequencing dynamics that are often central to interpretation. Additionally, these methods can lead to disproportionate mappings, where many points from one scanpath are matched to a small subset of points from the other, compromising the meaningfulness of the comparison.

Several refinements of the Mannan double-mapping approach have been proposed. For instance, the *EyeAnalysis* method (Mathôt et al., 2012) introduced a simplified and more adaptable similarity metric. This method calculates the sum of all point-mapping distances, normalized by the number of points in the longer sequence, ensuring that scanpaths of differing lengths are treated equitably. A key innovation in this approach is its incorporation of additional dimensions—such as timestamps and fixation durations—when determining optimal point pairings, providing a more comprehensive measure of similarity across spatial and temporal domains.

Henderson et al. (2007) further refined the Mannan metric by implementing a unique assignment procedure, enforcing a one-to-one mapping between fixation points. While this variant addresses issues of spatial variability and prevents over-mapping onto a limited subset of points, it is constrained to sequences of equal length and still fails to fully account for the temporal dynamics of fixation order. Paradoxically, this requirement for equal-length sequences contradicts the original motivation for the Mannan metric, which was designed to compare sequences of different lengths.

These limitations have motivated the development of more advanced comparison techniques that explicitly integrate the temporal dimension of scanpath sequences while maintaining flexibility in handling differences in length and complexity. Such methods, often framed as time-series alignment problems, represent a critical evolution in scanpath analysis, accommodating the multidimensional nature of eye-tracking data and advancing our ability to interpret visual behavior more comprehensively.

#### 3.1.2 Elastic alignment metrics

To address the limitations discussed in the previous section, researchers have increasingly turned to time-series alignment techniques that offer elastic measures of dissimilarity, such as *dynamic time warping* (DTW) and the *discrete Fréchet distance*. Both are widely used in time-series analysis across various fields and are particularly well suited for comparing trajectories that exhibit similar shapes but are not strictly time-synchronized.

**FIGURE 7:**
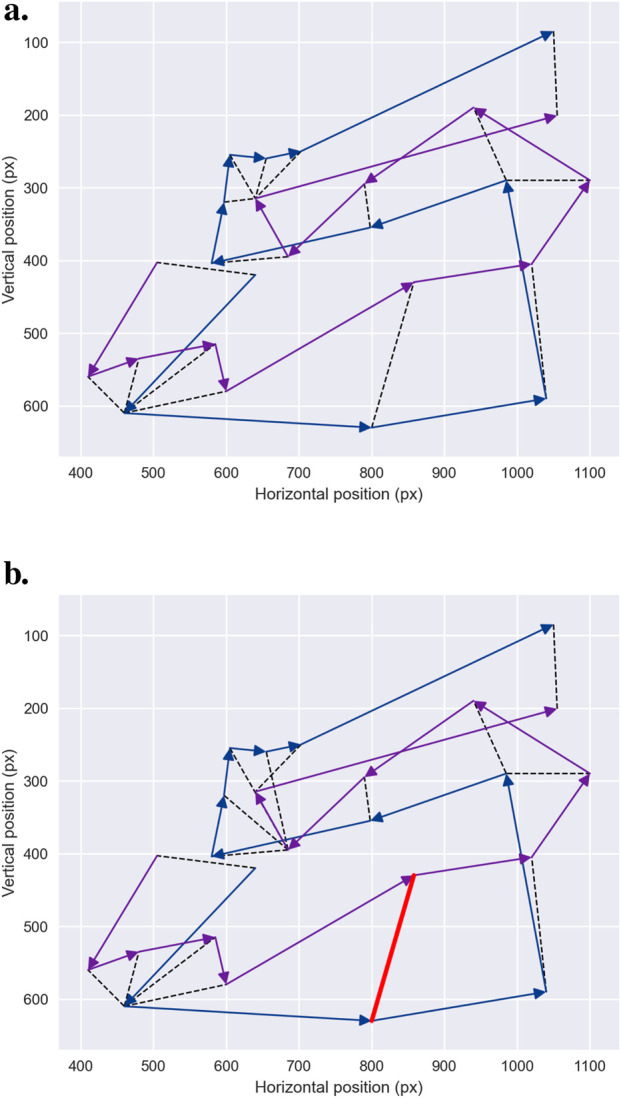
Elastic Metrics. Two scanpath trajectories—blue and purple curves—aligned using DTW and discrete Frechet distance. The DTW metric is computed by summing the length of all links between aligned data samples—figured by the black dotted lines. The Frechet distance, on the other hand, is calculated as the maximum distance—red line in **(b)** — between aligned data samples. **(a)** Dynamic time warping.

DTW compares two signals by aligning them in the time domain using dynamic programming. Initially introduced by Vintsyuk (1968) and Sakoe and Chiba (1978) for speech recognition, DTW measures the sum of the warps required to align one scanpath trajectory to another. Specifically, DTW seeks a temporal alignment—a mapping between time indices in the two series—that minimizes the Euclidean distance between aligned points. As a result, DTW provides a global measure of similarity that captures the overall shape and ordering of the trajectories, as illustrated in Figure 7. The key advantage of DTW lies in its ability to achieve robust time alignment between reference and test patterns, even when there are local accelerations or decelerations in the eye movement sequence (Brown et al., 2006).

**FIGURE 8:**
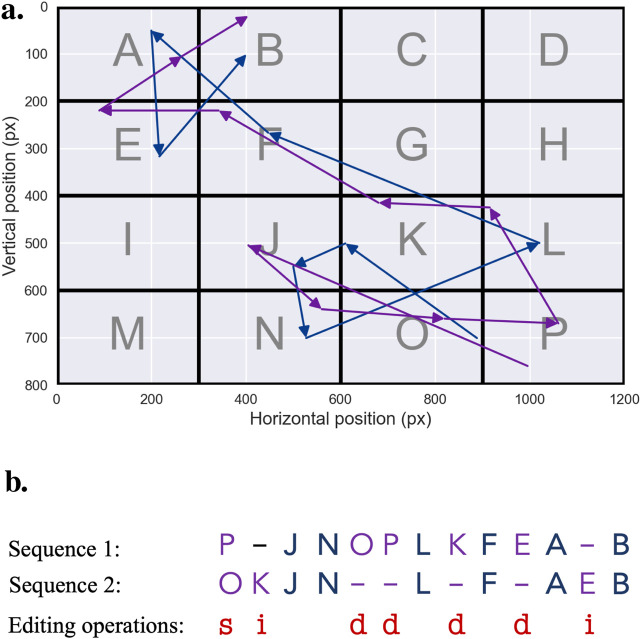
Levenshtein Edit Distance. The pairs of scanpaths to be compared—the purple and blue trajectories in **(a)** — are first converted into character sequences—for instance, in the example shown above, PJNOPLKFEAB and OKJNLFAEB . The resulting string sequences are then aligned — **(b)** — using the Wagner-Fischer algorithm and the minimum cost necessary to transform one sequence into another, using *insertions*, *deletions* and *substitutions* is computed. If *deletion* and *insertion* have cost of 1 and *substitution* a cost of 1.5, distance between the two scanpaths is 7.5.

The *discrete Fréchet distance* represents an alternative measure, distinct in its explicit penalization of temporal misalignments. The Fréchet distance can be intuitively understood as the shortest leash length required to connect two points: one moving along the first trajectory and the other along the second, where the points may travel at different rates but must move forward along their respective paths. Figure 7 illustrates this concept. The Fréchet distance provides a local measure of path similarity, focusing on the location and order of points while not allowing temporal indices to be arbitrarily warped. Like DTW, the discrete Fréchet distance is computed using dynamic programming (Eiter and Mannila, 1994).

Both DTW and the discrete Fréchet distance provide valuable measures of similarity. However, they also have important limitations that should guide their use. Unlike the Fréchet distance, DTW does not satisfy the triangle inequality and is therefore not a true distance metric. This limitation becomes particularly apparent when comparing scanpaths of different lengths, as DTW tends to overestimate the similarity between shorter and longer trajectories. Conversely, the discrete Fréchet distance is more sensitive to outliers and local deviations (Ahn et al., 2012). Despite these drawbacks, both DTW and the Fréchet distance are widely used in the literature to compare scanpaths without preprocessing (Le Meur and Liu, 2015; Li and Chen, 2018; Kumar et al., 2019), or as reference metrics to evaluate new methods (Wang et al., 2023). In applications involving large datasets, the computational cost of these alignment methods—and their scaling to pairwise distance matrices—should also be taken into account.

### 3.2 String edit distances

More than a single metric, the *string edit distance* encompasses a family of measures based on the concept of edit operations, enabling quantification of dissimilarity between sequences. In the context of scanpaths, these methods require converting fixation coordinates into string sequences, as detailed in Section 2.4. Once this transformation is performed, string edit distances can be applied to measure the similarity or divergence between scanpaths in a way that directly incorporates sequence order.

Among the various string edit distance methods, the *Levenshtein distance* (Levenshtein, 1966) remains one of the most frequently employed due to its simplicity and effectiveness (Holmqvist et al., 2011; Le Meur and Baccino, 2013). This approach calculates the minimum cost required to transform one sequence into another using three fundamental edit operations: (i) *deletion*, which removes an element from the string, (ii) *insertion* which adds an element into the string and (iii) *substitution* which replaces one element in the string with another. Each operation is assigned an edit cost, and the total transformation cost—usually computed using the Wagner–Fischer algorithm (Wagner and Fischer, 1974) — represents the Levenshtein distance between the two sequences. The Wagner–Fischer algorithm employs dynamic programming, iteratively computing a comparison matrix where rows correspond to the characters of one sequence and columns to those of the other. The algorithm determines the optimal alignment path through the matrix, with the distance given by the final matrix value. This score is often normalized by the length of the longer sequence to facilitate comparisons across scanpaths of differing lengths. The principle of scanpath comparison using the Levenshtein edit distance is illustrated in Figure 8, where two scanpaths are first converted into symbolic sequences and then optimally aligned using the Wagner–Fischer algorithm to compute the minimum transformation cost.

The Levenshtein distance has undergone substantial enhancements, with a variety of derivatives developed to improve both its accuracy and adaptability across diverse experimental contexts (Foulsham et al., 2008; Underwood et al., 2009; Harding and Bloj, 2010; Foulsham and Kingstone, 2013). While the original Levenshtein method remains effective, it traditionally assumes equal costs for all edit operations, disregarding factors such as the spatial proximity of fixation regions or their varying semantic significance. To overcome these limitations, recent adaptations have introduced variable weights for the *insertion* and *deletion* operations. Furthermore, many contemporary approaches incorporate a *substitution* cost function—typically represented as a substitution matrix—that accounts for the spatial relationships between different regions of the visual field. These enhancements facilitate a more nuanced and context-sensitive evaluation of scanpath similarity, allowing for a richer representation of meaningful patterns in fixation data (Josephson and Holmes, 2002a; Takeuchi and Habuchi, 2007; Takeuchi and Matsuda, 2012).

Additionally, alternative formulations of the string edit distance have been proposed. Notably, the *Damerau–Levenshtein distance* introduces a fourth operation, *transposition*, which swaps adjacent elements. This extension is especially beneficial when transpositions occur frequently in the data, as it reduces the overall edit distance in such cases (Foulsham et al., 2008). In contrast, the *longest common subsequence* (LCS) method focuses on local alignment by identifying the longest shared subsequence between two strings. LCS only considers *insertions* and *deletions*, excluding substitutions, providing a more intuitive measure of similarity based on common segments within the sequences. This approach is particularly valuable for detecting shared patterns in scanpaths, even when the sequences differ markedly in length or structure (Dewhurst et al., 2018; Davies et al., 2016; Eraslan and Yesilada, 2015).

Like any analytical method, string-edit distances have inherent limitations, primarily due to the spatial binning process used to discretize continuous scanpath trajectories into string sequences. This discretization can result in the loss of fine-grained spatial information, potentially limiting the method’s ability to capture detailed characteristics of the scanpath. The choice of grid resolution or AOI definition—and its interaction with the spatial structure of the stimulus—plays a central role in determining the sensitivity and interpretability of the resulting distances—see Section 2.4. Despite these limitations, string-edit distance remains a widely used and popular method for scanpath comparison, largely due to its simplicity, its clear link to sequence alignment, and the intuitive manner in which it quantifies dissimilarities between scanpaths. Furthermore, string-edit distance methods were foundational in early scanpath comparison research (Brandt and Stark, 1997) and have since been applied across a wide range of experimental contexts (Harding and Bloj, 2010; Underwood et al., 2009), making them particularly valuable for researchers seeking to compare their findings with previous studies. From a computational standpoint, classical string-edit distances scale quadratically with sequence length, which can limit their applicability to very long scanpaths or large pairwise comparison matrices without additional optimization.

### 3.3 Saliency comparison approaches

Saliency models, as discussed in Section 2.2.2, generate saliency maps that estimate the probability of different regions in an image attracting attention, thereby enabling automatic prediction of the most relevant areas. However, to validate these models across various applications or to quantify individual variations in gaze behavior, it is essential to analyze scanpaths derived from real data and apply appropriate comparison metrics.

In a similar vein, a *reference* saliency map—or *reference* attention map—can be constructed from the recorded fixations of a group of individuals, serving as a *ground truth* saliency map. A common task then involves comparing this reference saliency map with new scanpath recordings. To facilitate this comparison, we provide an overview of various metrics and analytical methods—often referred to as *hybrid* (Le Meur and Baccino, 2013) — for quantitatively comparing a saliency map with a single scanpath, and then turn to direct comparisons between pairs of saliency maps.

#### 3.3.1 Comparing reference saliency maps and scanpaths

A significant advantage of hybrid metrics is their ability to bypass the need for generating continuous saliency maps from fixation data, which often depend on parameterized models (Le Meur and Baccino, 2013). For instance, the choice of the Gaussian kernel’s standard deviation used to smooth fixation distributions introduces subjective decisions that can impact the results. By avoiding such dependencies, hybrid metrics provide a more direct and interpretable approach for assessing scanpath saliency when a reference map is available.

A first popular metric is the *normalized scanpath saliency* (NSS) introduced by Peters et al. (2005). To compute NSS, the reference saliency map is normalized by subtracting the mean saliency across all map locations and dividing by the standard deviation of saliency values, yielding a z -score. This z -score represents how many standard deviations the saliency value at a fixation point is above or below the average saliency. As human fixations typically do not align perfectly with individual pixels, NSS values for a fixation are calculated over a localized neighborhood centered around the fixation point (Le Meur and Baccino, 2013). This adjustment accounts for the spatial variability of human gaze, enhancing the robustness of NSS to minor positional discrepancies.

The *percentile* metric, introduced a few years later by Peters and Itti (2008), offers a straightforward yet effective means of quantifying the similarity between a viewer’s scanpath and a reference saliency map. For a given fixation, its associated saliency value is expressed as the proportion of map locations with lower saliency than at the fixation point. This percentile-based measure intuitively ranks each fixation’s saliency relative to the entire visual field. To compute a summary value for an entire scanpath, the individual saliency percentiles of all fixations are averaged. A key advantage of this approach lies in its simplicity and computational efficiency. Moreover, it is inherently invariant to re-parameterizations, as it relies on ranking saliency values rather than their absolute magnitudes, making it robust to monotonic transformations of the saliency map.

More recently, *information gain* (IG) was introduced by Kümmerer et al. (2014) and Kümmerer et al. (2015) as a robust metric to assess saliency model performance while accounting for systematic biases, such as the center prior. The center prior reflects the natural human tendency to fixate near the center of a visual scene, a phenomenon that can artificially inflate performance metrics for saliency models if not properly controlled. The information gain metric quantifies how much better a saliency model predicts recorded fixation points compared to a baseline model, typically the center prior. Mathematically, it measures the average increase in predictive power that the model offers over the baseline for the observed fixations. By focusing on the added predictive value beyond generic biases, IG provides a more nuanced evaluation of model performance, enabling researchers to isolate the unique contribution of a saliency model to fixation prediction.

Finally, it is essential to highlight location-based metrics, which are among the most extensively utilized measures for evaluating saliency maps (Bylinskii et al., 2018). These metrics are grounded in the concept of the area under the receiver operating characteristic curve (AUC), a widely applied tool in signal detection theory. AUC-based metrics evaluate the accuracy of a saliency map in predicting empirical fixations by interpreting the saliency map as a binary classifier, where each pixel is classified as either fixated or not fixated. The evaluation process begins by thresholding the *reference* saliency map—or *ground truth* saliency map—to retain a given percentage of the most salient pixels. By systematically varying the threshold, a *receiver operating characteristic* (ROC) curve is constructed, which plots the *true positive* rate—the proportion of correctly predicted fixated pixels—against the *false positive* rate—the proportion of non-fixated pixels incorrectly classified as fixated. The area under the ROC curve quantifies the overall prediction performance, with values closer to 1 indicating high predictive accuracy.

Several AUC implementations have been introduced, differing in how true positives and false positives are defined. A popular, straightforward approach called *AUC-Judd* (Judd et al., 2009; Bylinskii et al., 2014) computes true positive rates by considering the proportion of fixated pixels with saliency values exceeding a threshold, while false positive rates are derived from unfixated pixels exceeding the same threshold. Alternatively, *AUC-Borji* (Borji et al., 2012; 2013) employs uniform random sampling across the image to define false positives, improving robustness by controlling for uneven pixel distributions. Another variant, the *shuffled AUC* (sAUC), addresses the well-known center bias—the tendency of human observers to fixate near the center of visual stimuli—by using fixations from other images as the negative set, effectively sampling false positives predominantly from central regions of the image space (Zhang et al., 2008). Overall, location-based metrics provide an intuitive, flexible, and widely accepted framework for evaluating saliency models, balancing simplicity of computation with robust interpretability.

#### 3.3.2 Pair saliency comparison

Beyond hybrid approaches that compare fixation sets with reference saliency maps, a diverse range of methods has been developed for directly comparing pairs of saliency or attention maps. These methods provide complementary insights into the structural and statistical relationships between saliency distributions and are particularly useful when one wishes to compare two models, or two groups of observers, rather than individual scanpaths.

First, the *Kullback–Leibler divergence* (KL) is a key metric from information theory that quantifies the difference between two probability distributions (Kullback and Leibler, 1951). In the context of saliency maps, it evaluates how well an input saliency map approximates a reference map. Conceptually, it measures the information loss incurred when using the input distribution as a proxy for the reference. Lower KL divergence values indicate a closer match between the distributions. However, the asymmetry of KL divergence—requiring the designation of a reference map—and its unbounded upper limit can limit its intuitive interpretability and complicate comparative analyses across datasets. Despite these limitations, it remains a powerful tool for evaluating probabilistic saliency models (Rajashekar et al., 2004; Tatler et al., 2005; Le Meur et al., 2007) and can be adapted to compare pairs of maps generated by different models (Le Meur et al., 2006).

Another popular approach consists of using the *Pearson correlation coefficient* to quantify the strength of the linear relationship between two saliency maps. Widely adopted in computational models of visual attention (Jost et al., 2005; Le Meur et al., 2006; Rajashekar et al., 2008), this measure produces a single scalar value invariant to linear transformations, making it ideal for assessing overall alignment between maps. Values close to 1 signify a strong positive correlation, while values near −1 denote an inverse relationship. When a non-linear relationship is suspected, an alternative is the *Spearman rank correlation coefficient*, which assesses the relationship between the ranked values of two datasets (Toet, 2011). This rank-based approach provides robustness against non-linearities and outliers.

Finally, the *earth mover’s distance* (EMD) offers a spatially robust method to compare two saliency maps (Judd et al., 2012; Riche et al., 2013; Bylinskii et al., 2018). Unlike metrics that primarily assess value overlap, EMD quantifies the minimal effort required to transform one distribution into the other. This effort is computed as the product of the amount of density moved and the distance over which it is moved, effectively capturing spatial discrepancies between the maps. EMD thus addresses a key limitation of earlier methods—namely, the inability to account for small spatial misalignments. By incorporating positional differences into its calculations, EMD allows for a more nuanced comparison of maps, particularly in cases where distributions exhibit partial alignment or slight positional shifts in density. From a computational standpoint, metrics such as EMD and pixel-wise KL divergence can become costly for high-resolution maps or large numbers of pairwise comparisons, which should be considered when scaling saliency analyses to large datasets.

### 3.4 Cross recurrence quantification analysis

Beyond the comparison of single scanpaths or saliency maps, an increasingly influential line of work focuses on the temporal coordination between two observers or between an observer and a stimulus. In recent years, the adaptation of *cross recurrence quantification analysis* (CRQA) to scanpath comparison has generated a surge of research in gaze studies (Richardson and Dale, 2005; Richardson et al., 2009; 2007; Shockley et al., 2009; Cherubini et al., 2010; Dale et al., 2011a; b). CRQA extends the recurrence framework introduced in Section 2.3 to quantify dynamic coupling between two time series.

**FIGURE 9:**
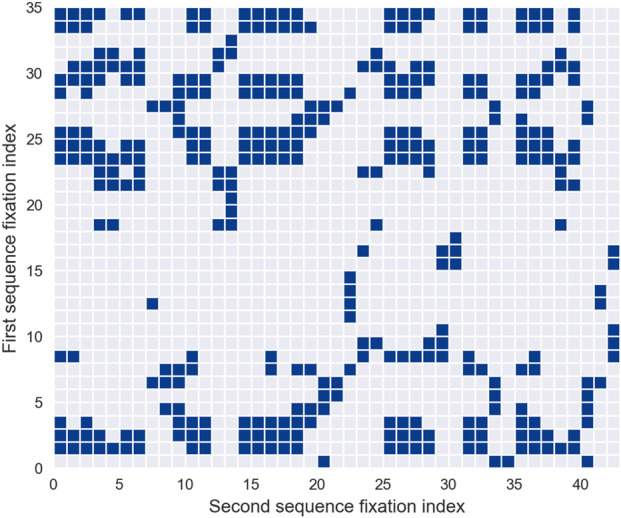
Cross Recurrence Quantification Analysis. A cross-recurrence plot is illustrated, with fixations from the first scanpath define the row divisions, while fixations from the second scanpath define the column divisions. A dot is placed at the (i,j) entry if the i -th fixation from the first scanpath is sufficiently close to the j -th fixation from the second scanpath. Similar to Recurrence Quantification Analysis (RQA), sets of diagonal and vertical lines can be extracted from the cross-recurrence plot to compute *cross-determinism* and *cross-laminarity*, respectively.

A *cross-recurrence plot* is essentially a matrix that visualizes the temporal coupling between two sequences of eye fixations. The vertical axis corresponds to the fixations of the first scanpath, while the horizontal axis represents the fixations of the second. Recurrence is indicated when two fixations, one from each sequence, fall within a predefined proximity radius. In the plot, recurrent pairs of fixations are represented as points, meaning the two systems exhibit similar states at corresponding times—see Figure 9. When the scanpaths are of equal length, points along the main diagonal of the recurrence plot represent synchronous recurrence—when the two viewers fixate on the same visual target at the same time. Points or diagonal lines offset from the main diagonal indicate recurring patterns with a time lag.

CRQA provides several metrics that can be assessed along the diagonal, horizontal, and vertical dimensions of the cross-recurrence plot. These metrics are adapted from the traditional RQA framework, but interpreted in the context of joint behavior (Anderson et al., 2015; Marwan et al., 2007). First, *cross-recurrence* quantifies the percentage of fixations that match between the two scanpaths. In essence, a higher cross-recurrence indicates greater spatial similarity between the two fixation sequences, reflecting their degree of spatial overlap in fixation locations.

In a manner similar to traditional RQA, *cross-determinism* measures the percentage of cross-recurrent points that form diagonal lines. These diagonal lines represent fixation trajectories that are shared by both sequences. This measure captures the overlap in specific fixation subsequences, preserving the temporal order of fixations. Cross-determinism is useful for identifying whether small subsequences of one scanpath are replicated in the other, even when the overall trajectories differ significantly.

Similarly, *cross-laminarity* quantifies repeated fixations in particular regions as the percentage of consecutive recurrence points in one fixation series that are aligned vertically with recurrence points in the other series, forming vertical structures in the combined recurrence plot. This measure is closely related to cross-determinism, and they are often interpreted together. High values of both cross-laminarity and cross-determinism suggest that both scanpaths tend to fixate on a few particular regions, with sustained fixation over several points in time. Conversely, a high cross-laminarity value coupled with low cross-determinism indicates that certain locations are explored in detail in one scanpath, but only briefly in the other.

Lastly, *cross-entropy* captures the complexity of the temporal coupling between two scanpaths by quantifying the variability of diagonal line lengths in the cross-recurrence plot. Low cross-entropy values indicate highly regular and stereotyped synchronization patterns, whereas higher values reflect more irregular, less predictable alignment between the two gaze sequences. In terms of computational complexity, CRQA relies on pairwise comparisons between complete scanpaths and therefore exhibits quadratic scaling with respect to scanpath length. As a result, the computational cost can become substantial for long recordings or large inter-observer datasets, unless strategies such as temporal windowing, sub-sampling, or parallelization are employed.

In some studies (Richardson and Dale, 2005; Shockley et al., 2009; Dale et al., 2011a; b), gaze data are quantified in terms of predefined *areas of interest* (AoIs). In this framework, two fixations are considered recurrent if they occur within the same AoI. Unlike traditional RQA, no spatial distance threshold needs to be set, as the cross-recurrence plot is reduced to a dot plot where fixations are marked as recurrent if they fall within the same predefined region. This approach emphasizes the semantic structure of the stimulus and its relation to joint attention. A more extensive discussion of AoI techniques and their methodological implications is provided in a separate dedicated contribution.

### 3.5 Specific comparison algorithms

The literature offers a diverse range of scanpath comparison algorithms, reflecting the depth and innovation within the field. Among these, three methodologies have emerged as particularly influential due to their widespread adoption and substantial contributions to scanpath analysis: *ScanMatch*, *MultiMatch*, and *SubsMatch*. These algorithms build on the representations and metrics discussed above, integrating them into cohesive frameworks that are well suited for practical applications and for deployment in software toolkits. The subsequent sections provide an overview of these approaches, highlighting their theoretical underpinnings, implementation techniques, and relative strengths.

#### 3.5.1 ScanMatch algorithm

Cristino et al. (2010) introduced the widely used *ScanMatch* method, a generalized approach for comparing scanpaths based on sequence alignment. ScanMatch provides a flexible framework for scanpath comparison by incorporating refined adaptations of the edit-distance methodology. The process begins with the transformation of input scanpaths into character strings, achieved through spatial and temporal binning of fixation sequences—see Section 2.4 for additional details.

The resulting character sequences are compared by maximizing a similarity score calculated using the Needleman–Wunsch algorithm. Similar to the Wagner–Fischer variants discussed in Section 3.2, Needleman–Wunsch employs dynamic programming to align two sequences. However, instead of merely penalizing divergent segments as in Wagner–Fischer, Needleman–Wunsch introduces matching bonuses for aligned segments, while negative matches are permitted when the segments exhibit a high degree of dissimilarity. The substitution matrix, central to this approach, encodes relationships between specific regions of the visual field, thereby tailoring the alignment process to the characteristics of the scanpath data.

The primary innovation of the ScanMatch method lies in the construction of the substitution matrix used to compare regions of the visual field. Traditionally, substitution matrices are based on the Euclidean distance between the centers of grid elements. However, Cristino and colleagues used the variability in saccade landing positions to determine a cutoff for assigning positive values in the substitution matrix—indicating highly related regions—and negative values for loosely related regions. The alignment algorithm is thus designed to match only those regions whose separation falls within the variability of saccade landing positions, with the threshold typically set to two standard deviations of the observed saccade amplitudes in a given experiment.

Ultimately, this method highlights the importance of carefully defining the substitution cost matrix between regions of the visual field. By introducing tolerance for variability in the mechanisms that control saccadic trajectories, ScanMatch overcomes many limitations of traditional editing methods. Additionally, it enables the incorporation of higher-order relationships between visual field regions. These relationships extend beyond spatial proximity and can also be defined by the semantic content of visual regions. This adaptability facilitates more nuanced and conceptually enriched similarity analyses, allowing for the consideration of a broader spectrum of contextual and interpretative factors.

#### 3.5.2 SubsMatch algorithm

*SubsMatch* is a string-based scanpath comparison algorithm designed by Kübler et al. (2014) to identify repeated patterns in visual behavior sequences. The method focuses on the computation of an extended transition matrix, which quantifies the occurrences of all subsequences of size n within a scanpath. Effectively, this approach can be viewed as a histogram-based method, where differences in occurrence frequencies serve as the foundation for evaluating similarity or dissimilarity between scanpaths.

The algorithm begins with a string-conversion process—see Section 2.4 — followed by the application of a sliding window of size n , which systematically counts the occurrences of all possible sub-sequences within the transformed string. This procedure generates a histogram representation, equivalently referred to as an n -gram embedding, which captures the frequency distribution of patterns of length n in the scanpath. This representation provides a detailed characterization of the scanpath’s structural features. Finally, the similarity between two scanpaths is assessed by evaluating the divergence between their sub-sequence frequency distributions.

This method has primarily been applied to compare eye movements associated with specific tasks (Braunagel et al., 2017a; b; Kübler et al., 2017). It was initially developed and validated in dynamic driving scenarios to distinguish between safe and unsafe driving behaviors (Kübler et al., 2014). More recently, SubsMatch has been utilized in diverse domains, such as identifying viewing behaviors that differentiate expert and novice micro-neurosurgeons, where it demonstrated significant group-level differences compared to other state-of-the-art metrics (Kübler et al., 2015).

An improved version of the algorithm, termed *SubsMatch 2.0*, was developed to address notable limitations of the original implementation (Kübler et al., 2017). One significant drawback of the initial approach was its uniform weighting of all sub-sequences, irrespective of their discriminative value. As a result, frequent yet non-informative patterns could exert undue influence on similarity scores. Furthermore, the initial algorithm relied on exact pattern matching, treating sub-sequences that differed by even a single substitution as entirely distinct, which limited its robustness in certain contexts. To address these issues, SubsMatch 2.0 introduced a classification-based methodology wherein sub-sequence frequency features were used as inputs to a support vector machine (SVM) with a linear kernel. This enhancement enabled the algorithm to assign greater importance to sub-sequences with higher discriminative value, improving its ability to differentiate between experimental conditions.

#### 3.5.3 MultiMatch algorithm

The *MultiMatch* algorithm (Dewhurst et al., 2012; Foulsham et al., 2012) introduces an alternative representation of scanpaths, modeling them as a series of concatenated saccade vectors. Each vector connects the coordinates of successive fixation points, encapsulating both the fixative and saccadic components of eye movements. The primary goal of the method is to achieve optimal alignment of these saccade vectors, enabling the extraction of meaningful metrics to compare the structural and temporal characteristics of scanpaths.

The process begins with a two-fold simplification step designed to reduce scanpath complexity through saccade clustering: (i) by combining into a single vector any two consecutive saccade vectors that are nearly collinear and (ii) by combining very short vectors with longer adjacent ones. These steps are applied iteratively until no further changes are observed, ensuring a progressive reduction in scanpath complexity. This approach enables the analysis of scanpaths that are too intricate to process directly, thereby enhancing computational feasibility. However, meticulous parameter selection and careful handling of the simplification process are crucial to maintaining the intrinsic characteristics of the original trajectories. The sensitivity of the outcomes to the chosen parameters underscores the importance of optimizing these settings for specific experimental contexts. By mitigating the influence of small saccades and localized fixations, the simplification step ensures that minor elements do not disproportionately bias similarity measurements. Once the scanpaths have been simplified, a temporal alignment process is performed to pair corresponding saccade vectors, enabling a robust and meaningful comparison of the scanpaths.

The alignment process, central to the algorithm, warrants further explanation. Initially, the norm of the vector difference between each saccade in the first scanpath and each saccade in the second scanpath is computed. These values are then stored in a *weight* matrix, which quantifies the shape similarity between all possible saccade pairings. Next, an *alignment* matrix is constructed, where the saccade vectors of the first scanpath are placed along the horizontal axis and the saccade vectors of the second scanpath along the vertical axis. This matrix defines the rules for allowed connections between vectors: connections are permitted only to the right, downward, or diagonally downward-right. Notably, backward connections are excluded, ensuring the alignment respects the temporal ordering of the scanpaths.

Together, the weight and alignment matrices form a directed, weighted graph. Nodes correspond to alignment matrix elements, edges represent permissible connections, and edge weights are defined by entries in the weight matrix. The optimal alignment is determined by finding the path through this graph that minimizes the total alignment cost. This is accomplished using Dijkstra’s algorithm (Cormen et al., 2022). Conceptually, this approach resembles *derivative dynamic time warping* (Eamonn and Michael, 2001), as highlighted by authors such as French et al. (2017), who suggested achieving alignment by minimizing cumulative differences using a vector difference matrix.

Once optimal alignment is established, several metrics can be extracted from the paired saccade vectors. This alignment allows for the comparison of both the saccadic and fixative components of the scanpaths—as mentioned earlier, the endpoints of saccade vectors correspond to fixation coordinates. More specifically, five commonly used similarity metrics can be derived from the alignment: (i) *shape* computed by determining the vector difference between aligned saccades, (ii) *length* which measures the similarity in saccadic amplitude, (iii) *position* which calculates the Euclidean distance between aligned fixations, (iv) *direction* which quantifies the angular difference between aligned saccade vectors and (v) *duration* which measures the difference in fixation durations between aligned fixations. Together, these metrics provide a comprehensive evaluation of both the saccadic and fixative aspects of the scanpaths, and they can be combined or analyzed separately depending on the research question.

### 3.6 Multi-scanpath comparison: towards group-level analyses

A central question, however, is how to interpret and use similarity and dissimilarity scores extracted from scanpaths. In practice, these scores are rarely meaningful in absolute terms; rather, they acquire interpretive value in comparative or inferential contexts. A common strategy is to evaluate whether within-participant similarity exceeds between-participant similarity, or whether scanpaths collected under a given experimental condition are more similar than those observed across conditions, typically using classical statistical procedures or permutation-based tests (Anderson et al., 2015). Closely related approaches rely on pairwise distance matrices computed across scanpaths, which can then be processed using clustering algorithms, multidimensional scaling, or supervised classification frameworks to reveal latent groupings, task-driven viewing strategies, or individual differences (Kumar et al., 2019; French et al., 2017; Castner, 2020). In all such applications, the interpretability of a metric depends on its sensitivity to spatial versus temporal structure, its robustness to noise and outliers, and its ability to scale to large collections of scanpaths.

Beyond pairwise comparison, several methodological traditions have emerged for multi-scanpath analysis. Some approaches derive group-level representations by aggregating information across observers, for instance through consensus-building procedures that estimate representative sequences or prototypical trajectories. Others emphasize the extraction of recurring subsequences, motifs, or transition structures across individuals, thereby shifting the analytical focus from global distance measures to shared structural patterns. A further class of methods adopts a graph-based perspective, representing gaze transitions as edges in a directed graph and comparing scanpaths through their transition dynamics or Markovian properties. Although these families of methods are often introduced in the context of raw, continuous scanpaths, they are conceptually much closer to the AoI-based approaches, where scanpaths are represented as sequences of discrete symbolic units. In practice, many of the multi-scanpath strategies outlined above—such as consensus-sequence construction, motif or subsequence extraction, and transition-based or graph-theoretic analyses—are more naturally, and more commonly, implemented on AoI sequences than on continuous fixation trajectories. This reflects a broader methodological point: most multi-scanpath comparison techniques implicitly rely on symbolization, discretization, and pattern extraction, all of which are foundational to AoI methodology.

For this reason, and to avoid redundancy, the detailed treatment of multi-scanpath approaches is deferred to a separate dedicated contribution focused on *Areas of Interest and Associated Algorithms*. There, these families of methods are revisited within their natural symbolic framework, allowing their assumptions, limitations, and interpretative affordances to be examined more thoroughly. By situating multi-scanpath comparison within the AoI paradigm, this symbolic perspective provides a more coherent and comprehensive account of the analytical tools that underpin group-level gaze analysis.

## 4 Discussion

The present review highlights both the methodological richness and the persistent fragmentation of the approaches used to characterize and compare scanpaths. Despite several decades of active research, scanpath analysis still lacks unified conceptual frameworks that clearly indicate *when* and *why* specific representations or metrics should be preferred. Scanpaths are inherently multidimensional entities, jointly embedding spatial, temporal, and semantic information. However, most existing methods focus on only one or two of these dimensions, and genuinely integrative approaches that account for the full complexity of the oculomotor signal remain relatively scarce.

A recurring challenge concerns the balance between intuitive, visually interpretable representations—such as scanpath plots, attention maps, or RQA recurrence plots—and more abstract quantitative metrics. Visual representations are accessible and powerful tools for exploratory analysis and qualitative comparison, particularly when multiple representations are shown side-by-side using the same gaze data. However, they provide only coarse-grained insight without formal quantification, and their interpretive value depends strongly on visualization choices, such as scale, grid resolution, or temporal sampling. This tension explains why many methods have evolved in parallel within the fields of visual analytics and information visualisation, a connection not always acknowledged in traditional eye-tracking literature but increasingly relevant for scanpath research.

From a quantitative perspective, the proliferation of available metrics reflects the diversity of research questions, but it also contributes to a degree of methodological opacity. Metrics differ widely in their sensitivity to spatial configuration, temporal order, noise, and outliers, and the interpretation of their absolute values is often non-trivial. In particular, certain conceptual interpretations require careful contextualization, especially in clinical settings where restricted visual exploration may reflect avoidance or impairment rather than efficiency or expertise. For these reasons, a more explicit discussion of interpretive limitations is essential for guiding both novice and advanced users. In the present review, emphasis is therefore placed on understanding most metrics as primarily descriptive tools, rather than as normative indicators of performance, efficiency, or optimality.

Beyond representational diversity, methodological choices such as grid size, discretization resolution, or segmentation parameters remain under-discussed in the literature, despite their substantial impact on results. For single and multi-scanpath analyses alike, these parameters determine whether subtle structure is preserved or lost. Similarly, scalability is an increasingly important concern: many classical comparison techniques were developed for pairwise comparisons and do not generalize efficiently to large datasets. As discussed in Section 3, more recent approaches leverage distance matrices, clustering algorithms, and supervised models to scale to dozens or hundreds of scanpaths, but their performance remains closely tied to representation choices and noise sensitivity.

Machine learning and deep learning approaches represent a promising response to several of the methodological challenges faced by classical scanpath analysis. By embedding scanpaths in high-dimensional feature spaces—through convolutional neural networks (CNNs), recurrent architectures, or more recent transformer-based models—these approaches can capture aspects of gaze behaviour that traditional metrics often overlook. For instance, Castner (2020) introduced an advanced variant of the string edit distance tailored specifically for scanpath analysis, in which the alignment cost between two fixations is computed from the norm of the difference between feature vectors extracted from the fixated image regions. These features are derived from a pre-trained CNN—specifically VGG-16 Simonyan and Zisserman (2014) — enabling the similarity measure to incorporate rich, high-level visual information rather than relying solely on geometric proximity.

In a broader application of deep learning, Ahn et al. (2020) investigated the classification of comprehension-related variables, including global text comprehension, passage-level understanding, and perceived reading difficulty. Their models relied directly on raw fixation coordinates and fixation durations, using both CNN and recurrent neural network (RNN) architectures to predict cognitive states from eye-tracking data. Together, these studies illustrate the potential of deep learning to infer complex cognitive variables directly from gaze behaviour.

Despite their promise, the performance and generalizability of learning-based approaches remain strongly constrained by the availability, quality, and diversity of training data. Human gaze behaviour exhibits substantial variability across individuals, tasks, stimuli, and viewing conditions, which complicates the construction of datasets that adequately capture this heterogeneity. Moreover, the collection of large-scale, well-annotated eye-tracking datasets remains costly and time-consuming, and dataset-specific biases can substantially affect model performance and transferability.

Recent advances in transfer learning (Rokni et al., 2018) and meta-learning (Gong et al., 2019) have partially alleviated these limitations by enabling models to adapt to novel tasks or domains from limited data. Nevertheless, their effectiveness still depends on the availability of robust and diverse base datasets for pre-training. To further mitigate data scarcity, generative modeling approaches have recently been proposed to synthesize large-scale, realistic eye-movement datasets. In particular, Lan et al. (2022) introduced a framework for generating synthetic scanpaths from publicly available images and videos, aiming to reproduce key statistical properties of human gaze while introducing variability across observers and experimental conditions. Although such synthetic data cannot yet fully replicate the complexity of human visual behaviour, they provide a scalable and controllable resource for training and benchmarking learning-based models.

Altogether, the integration of machine learning and deep learning into scanpath analysis marks a significant methodological shift. While these approaches introduce new challenges related to data heterogeneity, computational cost, and interpretability, ongoing progress in generative modeling, adaptive learning, and synthetic data generation offers promising avenues for overcoming these limitations. Ultimately, one of the most promising future directions lies in the development of hybrid frameworks that combine the interpretability of symbolic, AoI-based methods with the representational power of continuous, data-driven models, thereby enabling both robust quantitative analysis and meaningful cognitive interpretation.
